# TrypPROTACs Unlocking New Therapeutic Strategies for Chagas Disease

**DOI:** 10.3390/ph18060919

**Published:** 2025-06-19

**Authors:** Ana Luísa Rodriguez Gini, Pamela Souza Tada da Cunha, Emílio Emílio João, Chung Man Chin, Jean Leandro dos Santos, Esteban Carlos Serra, Cauê Benito Scarim

**Affiliations:** 1 Department of Drugs and Medicines, School of Pharmaceutical Sciences, Sao Paulo State University (UNESP), Araraquara 14800-903, SP, Brazil; ana.gini@unesp.br (A.L.R.G.); pamela.tada@unesp.br (P.S.T.d.C.); emilio.joao@unesp.br (E.E.J.); chungmanchin@gmail.com (C.M.C.); jean.santos@unesp.br (J.L.d.S.); 2 Facultad de Ciencias Bioquímicas y Farmacéuticas, Universidad Nacional de Rosario, Rosario S2000CGK, Argentina; estebancserra@gmail.com; 3 Instituto de Biología Molecular y Celular de Rosario (IBR), Consejo Nacional de Investigaciones Científicas y Técnicas (CONICET), Rosario S2000EZP, Argentina

**Keywords:** drug design, drug targeting, neglected tropical diseases, proteolysis targeting chimeras, *Trypanosoma cruzi* infection, ubiquitination

## Abstract

Chagas disease, caused by the protozoan parasite *Trypanosoma cruzi* (*T. cruzi*), continues to pose significant public health challenges due to the toxicity, poor tolerability, and limited efficacy of current treatments. Targeted protein degradation (TPD) using proteolysis-targeting chimeras (PROTACs) represents a novel therapeutic avenue by leveraging the ubiquitin–proteasome system to selectively degrade essential parasite proteins. This review introduces the conceptual framework of “TrypPROTACs” as a prospective strategy for *T. cruzi*, integrating a comprehensive analysis of druggable targets across critical biological pathways, including ergosterol biosynthesis, redox metabolism, glycolysis, nucleotide synthesis, protein kinases, molecular chaperones such as heat shock protein 90 (Hsp90), and epigenetic regulators such as *T. cruzi* bromodomain factor 3 (TcBDF3). It is important to note that no TrypPROTAC compound has yet been synthesized or experimentally validated in *T. cruzi*; the approach discussed herein remains theoretical and forward-looking. Representative inhibitors for each target class are compiled, highlighting potency, selectivity, and structural features relevant to ligand design. We also examine the parasite’s ubiquitination machinery and compare it to the human system to identify putative E3 ubiquitin ligases. Key aspects of linker engineering and ternary complex stabilization are discussed, alongside potential validation techniques such as the cellular thermal shift assay (CETSA) and bioluminescence resonance energy transfer (NanoBRET). Collectively, these insights outline a roadmap for the rational design of TrypPROTACs and support the feasibility of expanding targeted protein degradation strategies to neglected tropical diseases.

## 1. Chagas Disease: An Ongoing Public Health Challenge

Chagas disease, or American trypanosomiasis, is a neglected tropical disease caused by the protozoan *Trypanosoma cruzi* (*T. cruzi*) [1]. First described by Carlos Chagas in 1909, the disease follows a complex transmission cycle in which the parasite alternates between vertebrate hosts and insect vectors of the subfamily *Triatominae* (*Hemiptera: Reduviidae*) [2]. It is estimated that between six and seven million people are currently infected worldwide, with an increasing impact in non-endemic regions due to global migration and socioeconomic changes [3,4,5,6].

The primary route of *T. cruzi* transmission is vectorial, wherein infected triatomine insects excrete the parasite in their feces during blood feeding, allowing it to enter the host through microlesions in the skin or via mucosal contact [7,8,9]. However, transmission can also occur through non-vectorial pathways, including congenital transmission [10], ingestion of contaminated food [11], blood transfusion [12], and organ transplantation [13], complicating disease control and eradication efforts.

**Figure 1:**
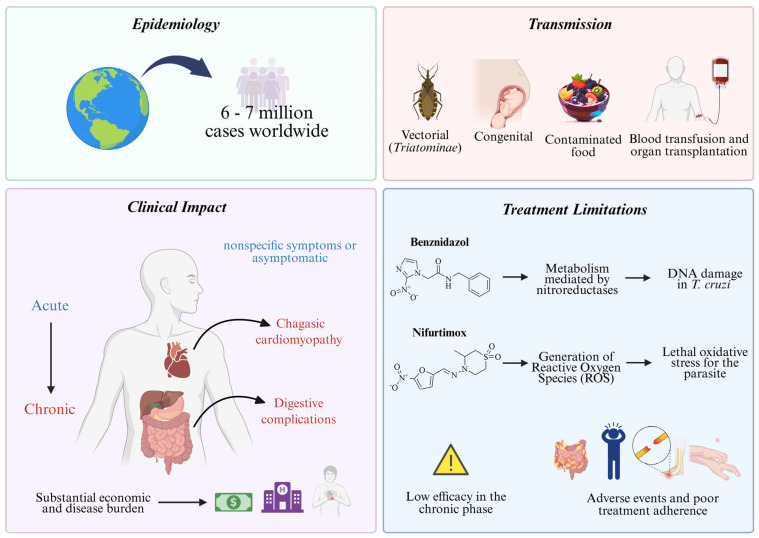
Overview of the epidemiology, transmission routes, clinical manifestations, and treatment limitations of Chagas disease. Created in BioRender. Dos santos, J. (2025) https://biorender.com/r22f256, accessed on 18 May 2025.

Clinically, Chagas disease progresses through two distinct phases [1]. The acute phase may be asymptomatic or present with nonspecific symptoms such as fever, lymphadenopathy, and hepatosplenomegaly, whereas the chronic phase can remain indeterminate for years or progress to severe complications [14,15,16]. Chagasic cardiomyopathy is the most serious chronic manifestation and represents a major cause of heart failure in Latin America. Additionally, digestive complications, such as megacolon and megaesophagus, significantly impact patient morbidity (Figure 1) [17,18,19].

Beyond its clinical burden, Chagas disease imposes substantial socioeconomic costs, which are reflected in high treatment expenses, reduced workforce productivity, and increased healthcare system demands [20,21,22]. Urbanization and the migration of infected individuals have expanded its geographic distribution, underscoring the need for improved screening programs in blood banks and maternal–infant healthcare services [23,24]. Concurrently, vector adaptation to peri-urban and sylvatic environments challenges conventional control strategies, necessitating integrated approaches for epidemiological surveillance and transmission prevention.

## 2. Current Treatment and Limitations

Chagas disease treatment remains limited to benznidazole (BZN) and nifurtimox (NFX), two drugs developed over 50 years ago [25,26]. Although effective in the acute phase, their efficacy in chronic infections is significantly reduced, and both are associated with a high incidence of adverse effects, leading to poor treatment adherence [27,28,29]. BZN is often the first-line option due to its relatively better tolerability compared to NFX, which generates reactive oxygen species (ROS) that induce oxidative stress and DNA damage in *T. cruzi* (Figure 1) [30,31]. Nevertheless, both drugs exhibit considerable toxicity, resulting in high discontinuation rates [32].

Approximately 40% of treated patients experience severe adverse reactions, including allergic dermatitis, peripheral neuropathy, and neuropsychiatric symptoms such as insomnia and irritability [33,34]. Furthermore, their limited efficacy against chronic infection remains a major concern, as intracellular *T. cruzi* amastigotes persist in host tissues, making parasite eradication particularly challenging [35].

To overcome these limitations, various strategies are under investigation, including drug repurposing, combination therapies, and the development of new nitro-heterocyclic derivatives [36,37]. Antifungal agents such as posaconazole and fosravuconazole have been evaluated; however, they did not achieve parasitological cure in clinical trials [38,39]. Fexinidazole, a structural analogue of BZN, has emerged as a potential alternative, though its clinical superiority over conventional treatments remains unproven [40,41]. In parallel, efforts have focused on optimizing treatment regimens to minimize adverse effects and improve adherence, with recent clinical studies suggesting that shorter treatment courses may reduce toxicity without compromising antiparasitic efficacy [42,43]. Despite these advancements, challenges remain, particularly the lack of reliable biomarkers to monitor therapeutic response and the difficulty in securing funding for large-scale clinical trials.

As research on *T. cruzi* advances, a deeper understanding of its biology has led to the identification of promising molecular targets, opening new avenues for the development of more selective and effective therapies [44,45]. Among the emerging strategies, targeted protein degradation (TPD) stands out as a particularly innovative approach for the treatment of Chagas disease [46]. In this context, PROTAC (proteolysis-targeting chimeras) technology—widely investigated in oncology—enables selective protein degradation by recruiting E3 ubiquitin ligases to direct specific proteins toward proteasomal elimination [47]. Applying this strategy to *T. cruzi* could transform therapeutic interventions by disabling essential parasite proteins. To fully exploit this potential, it is essential to deepen our understanding of *T. cruzi* morphology, its molecular targets, and the mechanistic principles of PROTAC technology, thereby laying the groundwork for the rational design of next-generation antiparasitic agents [46].

Although the term TrypPROTAC is introduced in this work to designate PROTAC-based strategies directed against *T. cruzi*, it is important to clarify that this concept remains at a theoretical stage. To date, no TrypPROTAC compound has been synthesized or experimentally evaluated in *T. cruzi*. Therefore, this review should be interpreted as a forward-looking perspective that outlines conceptual and methodological frameworks for future development.

## 3. Morphological Forms and Mitochondrial DNA Architecture in *T. cruzi*

Throughout its life cycle, *T. cruzi* presents three main developmental forms, which are epimastigote, amastigote, and trypomastigote [48,49]. The epimastigote is a replicative, flagellated form that proliferates in the midgut of the triatomine vector by binary fission [49,50]. Within mammalian cells, the parasite differentiates into the amastigote form, which is intracellular, rounded, and actively replicative [51]. The trypomastigote is an elongated, non-replicative, infective stage found in the insect’s excreta (metacyclic form) and in the host bloodstream [52]. This latter form is responsible for both host invasion and systemic dissemination. A key morphological distinction among these forms is the relative position of the kinetoplast, which is anterior to the nucleus in epimastigotes, posterior in trypomastigotes, and adjacent to the nucleus in amastigotes [48].

A hallmark of *T. cruzi* and other kinetoplastids is the presence of a single mitochondrion containing a condensed DNA-rich region termed the kinetoplast. This structure, located near the flagellar basal body, consists of a dense network of concatenated DNA molecules known as kinetoplast DNA (kDNA) [53,54,55]. In replicative forms, the kinetoplast appears as a concave disc of tightly packed DNA fibrils aligned along the longitudinal axis of the parasite, measuring approximately 1.0 µm in length and 0.1 µm in depth. It is separated from the inner mitochondrial membrane by a narrow space where cristae are often visible. In some cases, kDNA fibrils make direct contact with the mitochondrial membrane [56,57].

**Figure 2:**
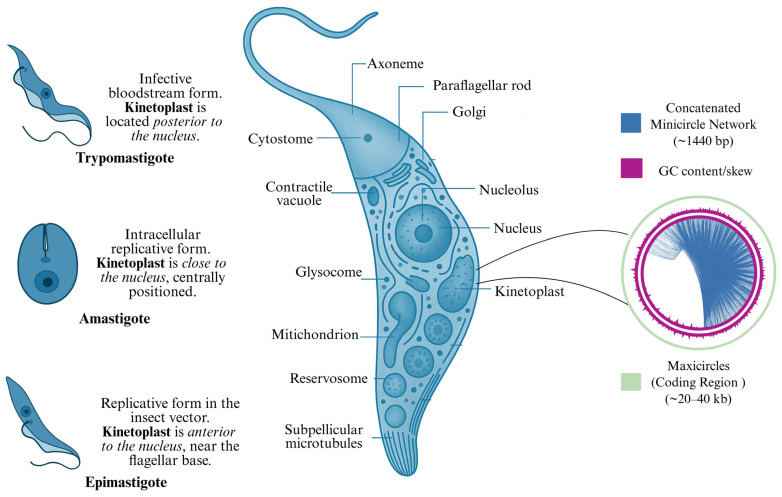
Morphological forms and mitochondrial DNA architecture of *T. cruzi.* Created in BioRender. Dos santos, J. (2025) https://biorender.com/r22f256, accessed on 18 May 2025.

The kDNA network comprises thousands of heterogeneous minicircles and a smaller number of maxicircles. Minicircles (~1440 base pairs) encode guide RNAs (gRNAs), which are essential for the RNA editing process that introduces uridine insertions and deletions into mitochondrial transcripts. Maxicircles (~20–40 kb) carry protein-coding genes such as *cytochrome b* and subunits of the mitochondrial ATP synthase, as well as ribosomal RNAs (12S and 9S). Only maxicircles are transcriptionally active, while minicircles provide the gRNA sequences necessary for the maturation of edited transcripts (Figure 2) [58,59,60].

Kinetoplast replication is tightly coordinated with the cell cycle. It begins with the duplication of the basal body and flagellum, followed by the elongation of the kDNA network. In some epimastigotes, the kinetoplast undergoes a characteristic transverse folding before division [61]. This intricate replication process reflects the structural and functional complexity of the kinetoplast and reinforces its relevance as a potential target for selective drug development.

## 4. Molecular Targets and Biological Pathways of *T. cruzi*

**Figure 3:**
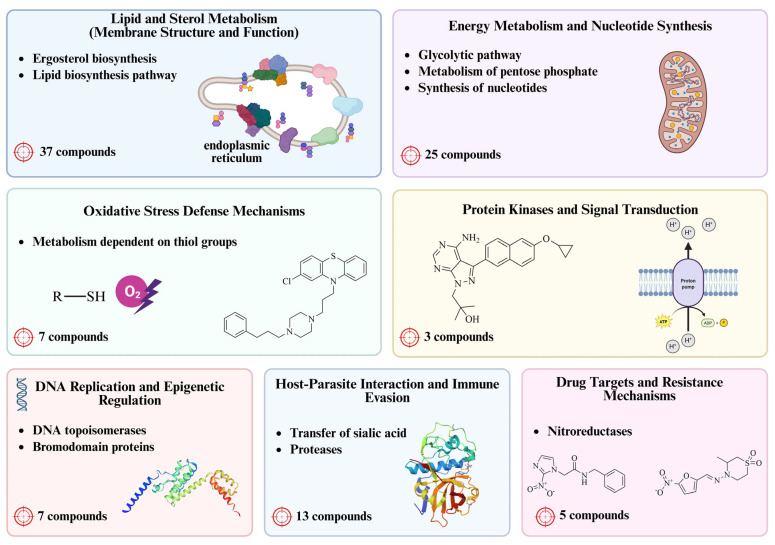
Biological pathways and molecular targets explored for drug discovery against *T. cruzi*. Created in BioRender. Dos santos, J. (2025) https://biorender.com/r22f256, accessed on 18 May 2025.

To support the rational identification of molecular targets in *T. cruzi* and guide the development of innovative therapeutic strategies such as PROTACs, we organized the parasite’s biological pathways into seven major categories. This classification aims to highlight the most relevant functional systems involved in parasite viability, infectivity, and survival. The seven categories considered in this analysis are as follows: lipid and sterol metabolism, energy metabolism and nucleotide synthesis, oxidative stress defense mechanisms, protein kinases and signal transduction, DNA replication and epigenetic regulation, host–parasite interaction and immune evasion, and drug targets and resistance mechanisms. This thematic organization facilitates a comprehensive and systematic exploration of promising targets for the development of targeted degradation strategies in *T. cruzi* (Figure 3).

Moreover, alongside the biological and mechanistic characterization, the lipophilicity of all tested compounds was estimated in silico to obtain LogP values. This physicochemical parameter was employed as an additional criterion to guide the selection of the most promising candidates for further discussion. LogP was considered in light of its potential impact on membrane permeability, target accessibility, and pharmacokinetic behavior, thus providing a complementary perspective for interpreting the observed biological activities.

### 4.1. Lipid and Sterol Metabolism (Membrane Structure and Function)

#### 4.1.1. Ergosterol Biosynthesis

The ergosterol biosynthesis pathway is essential for the survival of *T. cruzi*, as ergosterol plays a critical role in maintaining membrane integrity and fluidity. Unlike mammalian cells, which synthesize cholesterol, *T. cruzi* depends on ergosterol, providing a unique metabolic vulnerability that can be therapeutically exploited through the selective inhibition of key enzymes [62,63].

The early stages of this pathway include 3-hydroxy-3-methylglutaryl coenzyme A (HMG-CoA) reductase, farnesylpyrophosphate synthase (FPPS), squalene synthase (SQS), and squalene epoxidase (SE), leading to the formation of squalene epoxide. Inhibiting these enzymes disrupts sterol biosynthesis, resulting in ergosterol depletion and the accumulation of toxic intermediates, which ultimately compromises parasite viability [64,65].

Lanosterol synthase (LS) catalyzes the cyclization of squalene epoxide into lanosterol, a precursor of ergosterol. A critical downstream step is catalyzed by sterol 14α-demethylase (CYP51), which removes the 14α-methyl group from lanosterol. This enzyme is among the most extensively validated drug targets in *T. cruzi* due to its structural divergence from the human ortholog, allowing for the development of selective inhibitors with minimal off-target effects on host cholesterol metabolism [62,66,67].

Another promising target is Δ24(25)-sterol methyltransferase (24-SMT), which introduces a methyl group at a position not modified in human cholesterol biosynthesis. Inhibiting 24-SMT effectively blocks ergosterol production with high selectivity, representing an attractive therapeutic opportunity [68,69].

Beyond sterol biosynthesis, this pathway also produces isoprenoid intermediates necessary for protein prenylation, a post-translational modification essential for parasite survival. Farnesyltransferase (FTase), which catalyzes the transfer of farnesyl groups from farnesyl diphosphate synthase (FPP) to key regulatory proteins, is another potential target. Inhibitors of FTase or FPPS may interfere with essential signaling processes, offering complementary or alternative strategies to ergosterol-targeted interventions [70,71,72].

Despite their therapeutic potential, several enzymes in this pathway share homologs with human proteins, highlighting the need for highly selective inhibitors. Nevertheless, various compound classes, including nitrogen-containing bisphosphonates (targeting FPPS) and azoles (targeting CYP51), have demonstrated efficacy against *T. cruzi* [73,74]. Multitarget approaches that combine inhibitors acting on CYP51, FPPS, and FTase may enhance antiparasitic efficacy and reduce the risk of resistance development [64].

In summary, the ergosterol biosynthesis and isoprenoid pathways in *T. cruzi* encompass a variety of validated and emerging drug targets. The strategic combination of selective inhibitors targeting distinct enzymatic steps offers a promising avenue for the development of more effective therapies against Chagas disease [75].

Hurtado-Guerrero et al. (2002) investigated HMG-CoA reductase as a therapeutic target in *T. cruzi*, providing indirect biochemical evidence that statins inhibit the parasite’s mevalonate pathway [76]. Among the tested compounds, lovastatin (**1**) and simvastatin (**2**) exhibited the most potent activity against epimastigote forms cultured in sterol-free medium, with half-maximal inhibitory concentrations (IC_50_) of 0.3 µM and 0.4 µM, respectively. This inhibition was associated with a decrease in [^14^C]-acetate incorporation into sterols and an intracellular accumulation of HMG-CoA, suggesting functional enzyme blockade. Although the study did not include assays with the purified or recombinant enzyme, the findings support HMG-CoA reductase as a viable drug target and highlight the repurposing potential of statins as selective trypanocidal agents.

Montalvetti et al. (2001) cloned, expressed, and functionally characterized the farnesyl pyrophosphate synthase from *T. cruzi* (TcFPPS), validating it as a promising drug target [70]. They demonstrated that nitrogen-containing bisphosphonates, such as (**3**), (**4**), and especially (**5**), selectively inhibit TcFPPS activity. These compounds showed cytotoxic effects against intracellular forms of the parasite (IC_50_ values ranging from 60 to 147 µM) and in vivo efficacy, with risedronate being the most potent, likely due to the inhibition of sterol biosynthesis and preferential accumulation in parasite acidocalcisomes. Oliveira et al. (2018) conducted a hierarchical virtual screening combining pharmacophore modeling and molecular docking using the crystal structure of TcFPPS (PDB ID: 1YHL), leading to the identification of four promising natural product-derived candidates, which were (**6**), (**7**), (**8**), and (**9**) [77]. These compounds exhibited strong pharmacophore alignment, as indicated by QFIT scores above 60 (quantitative pharmacophore fit values), and favorable binding affinities, with docking scores (DS) ranging from −126 to −177 kcal/mol, closely mimicking the key interactions established by bisphosphonate inhibitors. Notably, (**6**) showed optimal interaction with the catalytic site, coordinating the three Mg^2+^ ions and forming stabilizing hydrogen bonds and π-cation interactions with conserved residues such as Asp98, Asp102, Asp250, Lys264, and Lys362, supporting its potential as a lead compound against *T. cruzi*. Romero-Solano et al. (2025) synthesized and evaluated Cu(II) complexes with risedronate as potential antiparasitic agents against *T. cruzi* and *Leishmania mexicana* (*L. mexicana*) [78]. Two complexes—(**10**) and (**11**)—stood out by significantly inhibiting the growth of *T. cruzi* epimastigotes in vitro, while maintaining low cytotoxicity toward COS-7 cells. Molecular docking studies revealed a strong affinity of both complexes for TcFPPS, with calculated binding free energies of −8.3 and −8.5 kcal/mol, respectively. The compounds established key interactions with catalytically relevant residues (Asp, Arg, Lys), suggesting possible competition with the enzyme’s natural substrates. Furthermore, the bisphosphonate moiety and metal coordination environment contributed to a spatial arrangement compatible with the TcFPPS active site. These findings support the hypothesis that TcFPPS inhibition may underlie the observed antiparasitic effects, positioning these Cu(II)–risedronate complexes as promising scaffolds for the rational development of selective TcFPPS inhibitors (Table 1).

Kamdem et al. (2023) provided a comprehensive review of SQS as an underexplored yet promising molecular target for anti-trypanosomatid drug discovery [79]. Focusing on *T. cruzi*, the authors emphasized the essential role of SQS in ergosterol biosynthesis—a pathway absent in humans—underscoring its potential for selective inhibition. From over 70 compounds compiled, more than 30 exhibited confirmed in vitro activity against trypanosomatids. Importantly, (**12**) and (**13**) demonstrated the direct enzymatic inhibition of TcSQS, with IC_50_ values of 6.9 and 6.4 nM for glycosomal SQS and 14.8 and 5.5 nM for microsomal/mitochondrial SQS, respectively [80]. These compounds also showed nanomolar potency against intracellular amastigotes (IC_50_ = 0.4–1.6 nM), and compound (**13**) achieved the complete suppression of parasitemia in vivo at 50 mg/kg for 30 days. Additionally, 2-alkylaminoethyl-1,1-bisphosphonates (compounds **14**–**19**) exhibited the potent enzymatic inhibition of TcSQS, with IC_50_ values ranging from 5 to 39 nM, as described by Rodríguez-Poveda et al. (2012) [81]. These findings validate TcSQS as a pharmacologically tractable target and provide a strong foundation for the structure-based optimization of selective inhibitors (Table 2).

Noguera et al. (2018) investigated a series of 4-arylthiazolylhydrazones derived from 1-indanones as potential inhibitors of TcSE, a key enzyme in the ergosterol biosynthesis pathway [82]. Experimental evaluation was based on squalene accumulation in protein-enriched cell-free extracts, supported by homology modeling of the enzyme, molecular docking, molecular dynamics simulations, and MM/PBSA binding free energy calculations. Compounds (**20**), (**21**), and (**22**) showed the most promising results, with squalene accumulation levels of 38–40%, IC_50_ values ranging from 4.27 to 4.53 µM, and binding energies between −5.83 and −8.63 kcal/mol. Additionally, (**22**) stood out as the most promising candidate, exhibiting the strongest theoretical binding affinity (−8.63 kcal/mol) along with high antiparasitic activity. Key interactions with residues Tyr105, Phe425, and Phe438 supported the proposed inhibition mechanism. These findings highlight these compounds as promising leads for the development of selective SE inhibitors against *T. cruzi* (Table 3).

Rabelo et al. (2017) reviewed rational design strategies for oxidosqualene cyclase (OSC) inhibitors, emphasizing its potential as a therapeutic target against trypanosomatids [83]. Among the compounds highlighted for TcOSC inhibition, compound (**23**) [84], an aminopropylindene analogue, exhibited an IC_50_ of 0.8 μM with low cytotoxicity (CC_50_ > 100 μM), while compound (**24**) [85], a phenolic ether of squalene, showed an IC_50_ of 1.1 μM. Umbelliferone derivatives were also effective, particularly *N*-oxides (**25**) and (**26**) [86] (IC_50_ ranging from 0.55 to 0.80 μM), suggesting that introducing electron-withdrawing groups may enhance interactions with the enzyme’s active site. Complementarily, Tani et al. (2018) developed a biochemical assay based on proton nuclear magnetic resonance (NMR) spectroscopy combined with factor analysis to quantify the activity of recombinant OSCs from *T. cruzi* and humans [87]. Compounds (**27**) and (**28**) exhibited potent inhibitory activity against the parasite OSC, with IC_50_ values of 42 and 44 nM, respectively. Notably, compound (**27**) also demonstrated efficacy against epimastigote forms (IC_50_ = 110 nM), whereas compound (**28**) showed greater selectivity for the parasite enzyme, presenting a selectivity ratio of 2.7, despite a modest reduction in cellular potency. These results further support OSC as a validated target for anti-trypanosomal drug development and underscore the potential of structurally distinct scaffolds with favorable inhibitory and selectivity profiles (Table 3).

**Table 3:** Selected SE and OSC inhibitors in *T. cruzi*: IC_50_ values, squalene accumulation, and binding affinities.

Compound	Structure	Affinity Data	LogP	Reference
**20**	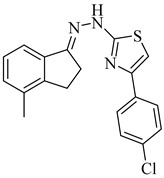	40.17% squalene accumulation	5.53432	[82]
**21**	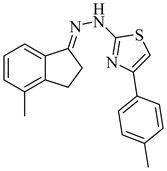	38.88% squalene accumulation	5.18934	[82]
**22**	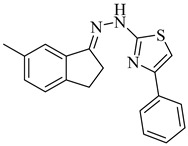	37.94% squalene accumulation	4.88092	[82]
**23**	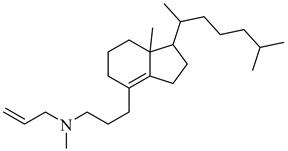	IC_50_ = 0.8 µM (TcOSC)	7.2436	[84]
**24**	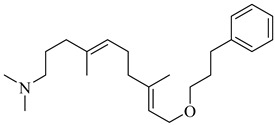	IC_50_ = 0.47 µM (TcOSC)	5.6504	[85]
**25**	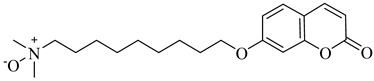	IC_50_ = 0.55 µM (TcOSC)	4.4767	[86]
**26**	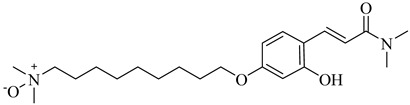	IC_50_ = 0.80 µM (TcOSC)	4.1773	[86]
**27**	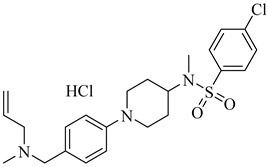	IC_50_ = 42 nM (TcOSC)	4.6691	[87]
**28**	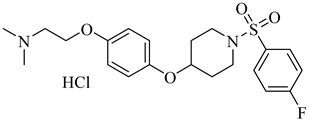	IC_50_ = 44 nM (TcOSC)	3.4200	[87]

Abbreviations: TcOSC = *T. cruzi* oxidosqualene cyclase; IC_50_ = half-maximal inhibitory concentration; LogP = octanol–water partition coefficient; squalene accumulation = intracellular accumulation of squalene, indicative of enzyme inhibition.

Building on the findings from Santos-Júnior et al. (2021) [88] and De Souza et al. (2023) [44], five azole and pyridine-based CYP51 inhibitors—compounds (**29**) [89] and (**30**) [90] from the former and compounds (**31**) [91], (**32**) [92], and (**33**) [93] from the latter—represent some of the most promising leads reported against TcCYP51. Compound (**29**) is a triazole derivative that exhibited an IC_50_ of 2 nM against intracellular amastigotes and an inhibition constant (*K*_i_) below 25 nM against TcCYP51, with a selectivity index (SI) of 2615 and 99.4% reduction of parasitemia in vivo. Compound (**30**) displayed subnanomolar potency in vitro, with half-maximal effective concentration (EC_50_) values ranging from 0.4 to 1.4 nM across independent assays and saturation of TcCYP51 binding at 2.2 µM, indicating a dissociation constant (*K*_d_) below this concentration. From the De Souza review, compound (**31**) exhibited an IC_50_ of 0.5 nM and *K*_d_ of 8.4 nM, with excellent oral tolerability. Compound (**32**) achieved an EC_50_ of 29 nM and *K*_d_ ≤ 10 nM, while compound (**33**) showed an EC_50_ of 0.4 μM and an IC_50_ 0.1 µM, with strong selectivity over human CYPs and co-crystallized binding to TcCYP51 (Table 4). All compounds operate via a type II inhibition mechanism, coordinating the heme iron and engaging key active-site residues such as Tyr103, Met106, and Phe290, contributing to their high binding affinity and mechanistic specificity. These compounds provide a chemically and mechanistically validated foundation for next-generation CYP51-targeted therapies for Chagas disease.

Urbina and collaborators (1995–1996) have extensively characterized 24-SMT as a selective target in *T. cruzi*, demonstrating that azasterol derivatives such as (**34**) potently inhibit the enzyme (>99% at 3 µM), disrupt parasite sterol biosynthesis, and induce growth arrest [69,94]. Follow-up studies confirmed that this inhibition leads to a shift from 24-alkylated sterols to non-alkylated intermediates, without altering total sterol content. Despite this compelling profile and the enzyme’s absence in humans, research on 24-SMT in *T. cruzi* has remained limited. Related investigations in *Leishmania* spp. [95] and *Trypanosoma brucei* (*T. brucei*) [68]—most notably by Urbina’s group and others—provided valuable biochemical and ultrastructural evidence of sterol pathway disruption; however, these studies are now considered somewhat dated. These cumulative findings underscore a persistent gap and support renewed efforts to exploit 24-SMT as a chemotherapeutic target for Chagas disease (Table 4).

Esteva et al. (2005) identified FTase from *T. cruzi* as a promising molecular target for antichagasic drug development due to its crucial role in the post-translational prenylation of CAAX-motif proteins, which is essential for parasite viability and signal transduction [96]. Guided by a homology model based on the rat FTase crystal structure, the authors designed benzophenone-based inhibitors with selective affinity for the parasitic enzyme. Enzyme inhibition assays using *Saccharomyces cerevisiae* FTase (ScFTase) highlighted two potent inhibitors, the *R*-phenylalanine derivative (**35**) and the *N*-propylpiperazinyl derivative (**36**), with IC_50_ values of 116 nM and 125 nM, respectively. Both compounds exhibited remarkable trypanocidal activity in vitro, with half-maximal lethal concentration (LC_50_) values of 1 nM (**35**) and 10 nM (**36**), and low cytotoxicity in mammalian cells (CC_50_ > 68 µM). These favorable profiles justified their selection for in vivo evaluation. Compound (**35**) demonstrated the highest SI (CC_50_ > 78 µM), whereas compound (**36**) combined nanomolar activity with enhanced aqueous solubility, likely due to the protonable *N*-propylpiperazine moiety. Upon oral administration in a murine model of acute *T. cruzi* infection, both compounds significantly improved survival rates (80% for **35** and 60% for **36**), reinforcing FTase as a validated yet underexplored therapeutic target for Chagas disease.

#### 4.1.2. Lipid Biosynthesis Pathway

The lipid biosynthesis pathway in *T. cruzi* displays distinctive biochemical features, notably the presence of ether-type phospholipids and glycosphingolipids containing inositolphosphorylceramide (IPC), which are absent in mammalian cells. These structural differences provide a rationale for exploring parasite-specific enzymes within these pathways as potential therapeutic targets. Nevertheless, this area remains underexplored in the context of Chagas disease, and key enzymes such as IPC synthase and alkyl-dihydroxyacetonephosphate synthase have not yet been functionally validated or targeted by selective inhibitors. As such, the lipid metabolic machinery of *T. cruzi* represents a promising yet still largely unexplored source of druggable targets [97,98,99].

Among the limited number of compounds evaluated in this context, miltefosine (**37**)—a synthetic alkyl-lysophospholipid—has demonstrated activity against both epimastigote and amastigote forms of *T. cruzi* [98]. Although it was not originally designed to interfere with specific lipid biosynthetic enzymes, functional studies suggest that (**37**) may impact lipid homeostasis by inhibiting phosphatidylethanolamine *N*-methyltransferase, thereby impairing phosphatidylcholine synthesis and membrane integrity. Additionally, the compound has been associated with perturbations in calcium homeostasis, further compromising organelle function. While these effects point to an indirect disruption of lipid-related processes, the precise molecular targets remain to be elucidated. As such, the lipid metabolic machinery of *T. cruzi* represents a promising yet still largely unexplored source of druggable targets (Table 4).

**Table 4:** Selected CYP51 and FTase inhibitors with potent in vitro activity and validated binding to the enzyme.

Compound	Structure	Affinity Data	LogP	Reference
**29**	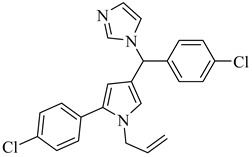	*K*_i_ < 25 nM (TcCYP51)	6.4821	[89]
**30**	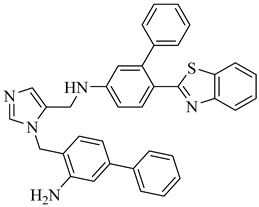	*K*_d_ < 2.2 µM (TcCYP51)	8.7364	[90]
**31**	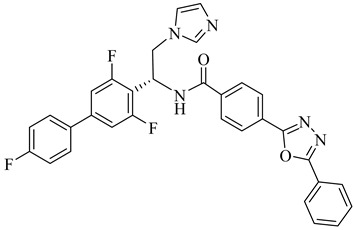	*K*_d_ = 8.4 nM (TcCYP51)	6.8557	[91]
**32**	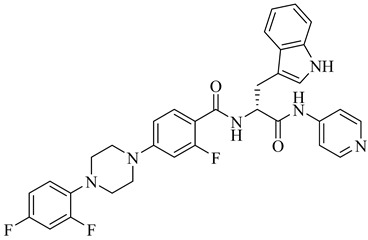	*K*_d_ < 10 nM (TcCYP51)	5.2866	[92]
**33**	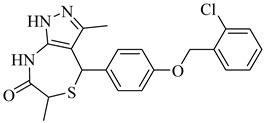	IC_50_ = 0.1 µM (TcCYP51)	5.11372	[93]
**34**	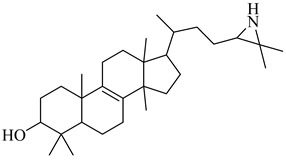	>99% inhibition (at 3 µM) (Tc24-SMT)	7.2633	[94]
**35**	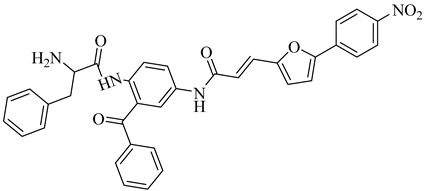	IC_50_ = 116 nM (ScFTase)	6.2462	[96]
**36**	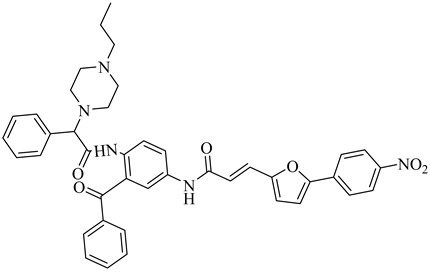	IC_50_ = 125 nM (ScFTase)	7.4451	[96]
**37**	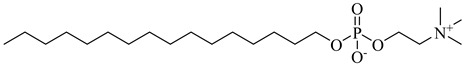	—	5.6755	[98]

Abbreviations: TcCYP51 = *T. cruzi* cytochrome P450 51; Tc24-SMT = *T. cruzi* sterol 24-C-methyltransferase; ScFTase = *S. cerevisiae* farnesyltransferase; IC_50_ = half-maximal inhibitory concentration; *K_i_* = inhibition constant; *K_d_* = dissociation constant; LogP = octanol–water partition coefficient.

### 4.2. Energy Metabolism and Nucleotide Synthesis

#### 4.2.1. Glycolytic Pathway

##### Glyceraldehyde-3-Phosphate Dehydrogenase

The glyceraldehyde-3-phosphate dehydrogenase from *T. cruzi* (TcGAPDH) is an essential enzyme in the glycolytic pathway and has emerged as a promising therapeutic target for Chagas disease [100]. Its catalytic function involves the conversion of glyceraldehyde-3-phosphate (G3P) into 1,3-bisphosphoglycerate with the concomitant reduction of NAD^+^ to NADH, which is critical for ATP generation in the epimastigote and amastigote forms of the parasite. The crystal structure of TcGAPDH (PDB: 1QXS) reveals key catalytic residues such as Cys166, Thr197, Thr199, Arg249, and Ser247, which differ significantly from those in the human isoform (hGAPDH), supporting the design of selective inhibitors [101,102].

Beyond its glycolytic role, TcGAPDH is a multifunctional (“moonlighting”) protein with nuclear functions linked to genome integrity. It has been shown to associate with single-stranded telomeric DNA in the replicative forms of the parasite via its NAD^+^-binding domain. This interaction is regulated by the intracellular NAD^+^/NADH ratio, as high NADH levels promote telomeric association, while elevated NAD^+^ concentrations, as observed in trypomastigotes, inhibit the interaction and induce the relocalization of the enzyme to the nucleolus. This redox-dependent behavior has been confirmed by electrophoretic mobility shift assay and chromatin immunoprecipitation assays. This mechanism suggests that TcGAPDH protects telomeres during parasite proliferation, integrating metabolic and nuclear functions [103,104]. Therefore, TcGAPDH stands out as a pharmacological target with multiple intervention possibilities, either by inhibiting its essential enzymatic activity or by modulating its nuclear roles related to chromosomal stability and stress response.

In 2010, Silva et al. explored TcGAPDH as a potential therapeutic target and evaluated ruthenium-based nitric oxide donor complexes for their trypanocidal activity [105]. Three compounds—(**38**), (**39**), and (**40**)—showed inhibitory activity against TcGAPDH with IC_50_ values ranging from 89 to 153 µM, acting through irreversible *S*-nitrosylation of the catalytic Cys166 residue. Beyond enzyme inhibition, these complexes demonstrated significant trypanocidal effects both in vitro and in vivo, including reduced parasite burden in cardiac tissue and increased survival rates in infected mice. Later, in 2014, Prokopczyk et al. [106] employed structure- and ligand-based virtual screening approaches, followed by isothermal titration calorimetry (ITC), to identify selective TcGAPDH inhibitors. From a library of 25 commercially available compounds, three hits stood out—(**41**), (**42**), and (**43**)—all displaying apparent *K*_i_ values below 100 µM. Among them, (**41**), a dihydroindole derivative, emerged as the most promising candidate, acting as a competitive inhibitor at the G3P binding site, while sparing the NAD^+^ binding region, suggesting favorable selectivity toward the parasitic enzyme (Table 5).

##### Hexokinase/Glucokinase

The glycolytic metabolism of *T. cruzi* critically depends on the enzymes glucokinase (TcGlcK) and hexokinase (TcHxK), which catalyze the phosphorylation of glucose to glucose-6-phosphate (G6P). This metabolite plays a central role in the parasite’s energy metabolism, fueling pathways such as glycolysis and the pentose phosphate pathway, which are essential for ATP production, redox balance—mediated by nicotinamide adenine dinucleotide phosphate (NADPH)—and nucleotide biosynthesis [107,108]. Inhibiting these enzymes can directly impair glycolytic flux and redox homeostasis, making them promising targets for the development of new chemotherapeutics against Chagas disease [109].

Although TcGlcK and TcHxK catalyze the same reaction, they differ significantly in structure, biochemical properties, and regulation. TcHxK is more abundant in the parasite, exhibits higher glucose affinity—reflected by a low Michaelis–Menten constant (*K*_m_ ≈ 0.06 mM)—is inhibited by G6P, and shows hysteresis behavior, suggesting allosteric regulation. In contrast, TcGlcK has a lower affinity (*K*_m_ ≈ 1.0 mM), is not inhibited by G6P, and has been crystallized as a functional dimer with a hydrophobic intersubunit pocket that accommodates aromatic inhibitor moieties [110,111]. The coexistence of these two non-homologous glucose kinases—a rare feature among protozoa—strengthens the rationale for targeting them individually or simultaneously [112]. The rational exploration of their structural distinctions may be decisive for designing selective and effective inhibitors, overcoming the limitations of currently available treatments (Table 6).

In 2015, D’Antonio et al. applied a structure-based drug design (SBDD) approach to develop selective glucosamine-based inhibitors of TcGlcK [113]. Among the tested compounds, (**44**) emerged as the most potent TcGlcK inhibitor to date, with a *K*_i_ of 0.71 µM and a SI of 245 over human HxK IV. Other analogues, such as (**45**) and (**46**), also exhibited strong inhibition (*K*_i_ = 1.3 and 4.2 µM, respectively), whereas (**47**) demonstrated moderate enzymatic potency (*K*_i_ = 32 µM) alongside unexpectedly strong antiparasitic activity (IC_50_ = 16.1 µM). The authors suggested that compound (**47**) may act on both TcGlcK and TcHxK, potentially due to physicochemical properties that favor dual engagement and glycosomal uptake. These results established key chemical scaffolds for targeting *T. cruzi* glucose kinases, including potential dual-target inhibitors. In 2019, Mercaldi et al. conducted a high-throughput screening (HTS) of 13,040 compounds, aiming to identify selective inhibitors of TcGlcK [114]. This screening led to the identification of (**48**) and (**49**), both belonging to the 3-nitro-2-phenyl-2*H*-chromene class, which exhibited potent TcGlcK inhibition (IC_50_ = 6.1 µM and 4.8 µM, respectively), low-micromolar antiparasitic activity (EC_50_ = 2.1 and 2.9 µM), and low cytotoxicity in NIH-3T3 cells. In 2024, Carey et al. expanded the biochemical characterization of this series, confirming that (**48**) acts as a noncompetitive TcGlcK inhibitor (*K*_i_ = 6.2 µM), while a related analogue (**50**) exhibited mixed inhibition (*K*_i_ = 5.5 µM) [115]. Both retained high *T. cruzi* activity and favorable selectivity profiles. When integrating findings from both studies, (**48**), (**49**), and compound (**50**) emerged as the most promising hit-to-lead candidates of the series, combining potent enzymatic inhibition, cellular efficacy, and host cell selectivity. These compounds represent valuable scaffolds for further optimization toward novel TcGlcK-targeted therapies for Chagas disease (Table 6).

##### Phosphofructokinase

In *T. cruzi*, PFK catalyzes the irreversible conversion of fructose-6-phosphate to fructose-1,6-bisphosphate, playing a central role in glycolysis [116]. Due to its compartmentalization within glycosomes and its central role in ATP production, PFK has emerged as an attractive therapeutic target, especially considering the parasite’s heavy reliance on glycolysis. Biochemical studies have revealed unique kinetic properties, including allosteric activation by adenosine monophosphate and inhibition by fructose-1,6-bisphosphate, as well as a high affinity for ATP [117]. Building upon these biochemical characteristics, several selective inhibitors have been developed—most notably the para-amidosulfonamide derivatives—which exhibit nanomolar potency against TcPFK while sparing the human isoform.

Brimacombe et al. (2014) reported the identification of selective small-molecule inhibitors targeting phosphofructokinase (PFK) from *T. cruzi*, a critical glycolytic enzyme essential for parasite survival [118]. Through a quantitative high-throughput screening campaign and subsequent structure–activity relationship (SAR) optimization, the authors identified the *para*-amidosulfonamide scaffold as a potent chemotype. Further analogues demonstrated enhanced potency, especially compound (**51**) and compound (**52**), which exhibited IC_50_ values of 41 nM and 52 nM, respectively, against TcPFK. Mechanistic studies revealed that these compounds competitively inhibit fructose-6-phosphate binding and act as mixed inhibitors with ATP, suggesting interaction with the enzyme’s active site in its open conformation. Both compounds showed excellent selectivity over mammalian PFK isoforms and no cytotoxicity in human MRC-5 cells at concentrations up to 46 µM. These results position compounds (**51**) and (**52**) as potent and selective enzymatic inhibitors of *T. cruzi* PFK, supporting their further development as chemical probes or antiparasitic lead candidates (Table 6).

#### 4.2.2. Metabolism of Pentose Phosphate

##### 6-Phosphogluconate Dehydrogenase

6-Phosphogluconate dehydrogenase (6PGDH) functions within the oxidative phase of the pentose phosphate pathway, generating NADPH that is critical for sustaining redox balance via the trypanothione-dependent antioxidant system in *T. cruzi*. The enzyme is essential for parasite survival under oxidative stress and differs structurally from its human counterpart, offering potential for selective inhibition. The *T. cruzi* 6PGDH displays limited in vitro stability due to the absence of key inter-subunit salt bridges. A recombinant double-mutant version (V244D/C257R) restored dimer stability and retained native-like kinetics (*K*_m_ = 22.2 µM for 6PG; 5.9 µM for NADP^+^), enabling its use in screening assays [119,120].

Despite its druggability and metabolic relevance, no inhibitors have been specifically developed for the *T. cruzi* enzyme. Compound (**53**), a potent inhibitor of the *T. brucei* orthologue (*K*_i_ = 0.16 µM), has not yet been evaluated against the corresponding enzyme in *T. cruzi* [121]. The lack of chemical matter may reflect historical challenges in enzyme handling rather than intrinsic limitations of the target (Table 6).

**Table 6:** Targeting glycolysis and pentose phosphate pathways in *T. cruzi*.

Compound	Structure	Affinity Data	LogP	Reference
**44**	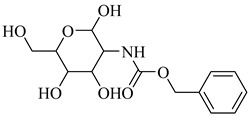	*K*_i_ = 0.71 µM (TcGlcK)	−1.2873	[113]
**45**	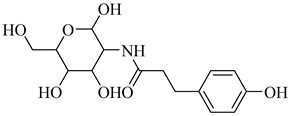	*K*_i_ = 1.3 µM (TcGlcK)	−1.7591	[113]
**46**	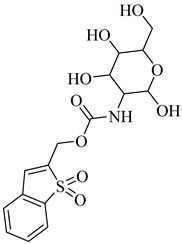	*K*_i_ = 4.2 µM (TcGlcK)	−1.6591	[113]
**47**	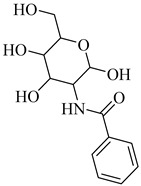	*K*_i_ = 32 µM IC_50_ = 16.1 µM (TcGlcK)	−1.7837	[113]
**48**	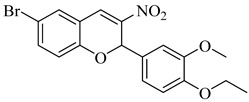	*K*_i_ = 6.2 µM (TcGlcK)	4.6077	[114]
**49**	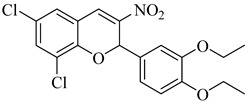	*K*_i_ = 4.8 µM (TcGlcK)	5.5421	[114]
**50**	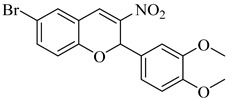	*K*_i_ = 5.5 µM (TcGlcK)	4.2176	[115]
**51**	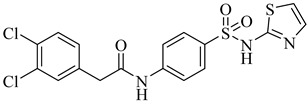	IC_50_ = 0.041 µM (TcPFK)	2.498	[118]
**52**	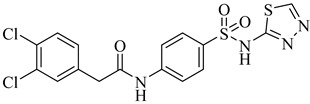	IC_50_ = 0.052 µM (TcPFK)	2.2904	[118]
**53**	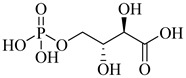	*K*_i_ = 0.16 µM (*T. brucei* 6PGDH)	−2.0979	[121]

Abbreviations: TcGlcK = *T. cruzi* glucokinase; TcPFK = *T. cruzi* phosphofructokinase; *T. brucei* 6PGDH = *Trypanosoma brucei* 6-phosphogluconate dehydrogenase; IC_50_ = half-maximal inhibitory concentration; *K*_i_ = inhibition constant; LogP = octanol–water partition coefficient.

#### 4.2.3. Synthesis of Nucleotides

##### Purine Phosphoribosyltransferases

*T. cruzi* relies entirely on the purine salvage pathway to meet its nucleotide demands, due to the absence of de novo purine biosynthesis. In this context, purine phosphoribosyltransferases (PRTs) play a central role by converting free purine bases into monophosphate nucleotides using 5-phospho-α-D-ribose 1-pyrophosphate (PRPP) as the ribose-phosphate donor [122,123]. This metabolic dependency highlights PRTs as promising targets for therapeutic intervention.

The *T. cruzi* genome encodes four distinct PRT isoforms, including two hypoxanthine-guanine phosphoribosyltransferases (HGPRTs, TcA and TcC) and two hypoxanthine-guanine-xanthine phosphoribosyltransferases (HGXPRTs, TcB and TcD). While HGPRTs preferentially act on hypoxanthine and guanine, HGXPRTs exhibit high specificity for xanthine [123,124]. This diversity suggests functional specialization and adaptation to different host environments or life cycle stages, supporting the rationale for targeting multiple isoforms simultaneously.

In a recent study, Glockzin et al. (2023) evaluated a panel of immucillin-based transition-state analogues as inhibitors of PRTs from *T. cruzi* [124]. Among the compounds tested, three analogues demonstrated notable potency against both TcHGXPRT and TcHGPRT, which were (**54**), (**55**), and (**56**). Compound (**54**) was the most potent dual inhibitor, with *K*_i_ values of 0.6 µM for TcHGXPRT and 0.006 µM for TcHGPRT. Compound (**55**) showed exceptional activity against TcHGPRT (*K*_i_ = 0.004 µM), although it was slightly less active against TcHGXPRT (*K*_i_ = 1.6 µM). Compound (**56**) exhibited balanced inhibition, with *K*_i_ values of 0.7 µM for TcHGXPRT and 1.4 µM for TcHGPRT (Table 7). These analogues mimic the enzymatic transition state and establish favorable interactions within the active site, stabilizing the enzyme–inhibitor complex via contacts with PRPP and the purine-binding region.

##### Dihydrofolate Reductase

The enzyme dihydrofolate reductase (DHFR) from *T. cruzi* (TcDHFR), part of a bifunctional protein together with thymidylate synthase, plays a critical role in the regeneration of tetrahydrofolate, an essential cofactor in nucleotide and amino acid biosynthesis. Unlike the monofunctional human DHFR, TcDHFR exhibits distinct structural features, including the presence of Met49 in the active site—replacing the corresponding Phe31 in the human enzyme—thus offering a valuable opportunity for selective inhibitor development [125,126].

These structural differences support the exploration of TcDHFR as a druggable target, with potential for the rational design of selective inhibitors using pharmacophore-based strategies and 3D-QSAR (quantitative structure–activity relationship) modeling. Despite the overall conservation of DHFR architecture across species, local variations in the active site and ligand-induced conformational flexibility justify structure-guided approaches aimed at improving selectivity and minimizing off-target toxicity [127].

Schormann et al. (2008) provided the first high-resolution structural data for *T. cruzi* dihydrofolate reductase–thymidylate synthase (DHFR–TS), a bifunctional enzyme that plays a central role in folate metabolism and DNA synthesis, thereby enabling structure-based drug design via crystallography, pharmacophore modeling, and 3D-QSAR analyses [127]. Using a set of 30 inhibitors, including trimetrexate (TMQ), the authors identified conserved molecular interactions and confirmed the enzyme’s tractability as a drug target. Building on these insights, Schormann et al. (2010) synthesized six novel TMQ analogues based on a 2,4-diaminoquinazoline scaffold [128]. These compounds inhibited the *T. cruzi* enzyme with nanomolar potency (IC_50_ = 23.8–57.5 nM; K_i_ = 1.3–3.3 nM). Notably, compound (**57**) was co-crystallized with DHFR–TS in complex with NADPH, revealing critical contacts with residues Asp48, Val26, and Met49. Although these interactions—especially hydrophobic contacts—were absent in the human orthologue (hDHFR), all compounds also inhibited the host enzyme, resulting in modest selectivity indices (SI = 2.9–5.8) (Table 7). These findings reinforce the therapeutic relevance of *T. cruzi* DHFR–TS while also illustrating the challenge of achieving parasite-specific inhibition.

##### Pteridine Reductase

Pteridine reductase 1 (PTR1) is a short-chain dehydrogenase/reductase family enzyme that is NADPH-dependent and present in *T. cruzi*. It catalyzes the reduction of biopterin to dihydrobiopterin (H_2_B), which is subsequently converted into tetrahydrobiopterin (H_4_B) [129]. PTR1 is also capable of reducing folate to dihydrofolate (H_2_F), although with lower affinity. The enzyme functions as a homotetramer, with four active sites and NADPH-binding regions. Each subunit features an α/β-domain with a Rossmann-fold architecture, consisting of seven parallel β-sheets flanked by α-helices [130]. In *T. cruzi*, the isoforms TcPTR1 and TcPTR2 share high sequence identity; however, they exhibit distinct enzymatic profiles, with TcPTR1 showing greater activity toward biopterin and folate. As the crystal structure of TcPTR1 has not yet been elucidated, homology models based on the structure of TcPTR2 have been employed in structural and molecular modeling studies [131].

In the study conducted by Cavazzuti et al. (2008), a targeted screening of folate analogues led to the identification of selective inhibitors of PTR1 from *T. cruzi*, a key enzyme involved in the parasite’s folate salvage pathway [132]. Among the tested compounds, derivative (**58**), a 2,4-diaminopteridine, emerged as the most promising candidate, exhibiting a *K*_i_ of 7 µM against TcPTR1 while maintaining favorable selectivity over human DHFR. Importantly, compound (**58**) demonstrated intracellular antiparasitic activity by inhibiting 22% of amastigote growth at 50 µM, supporting its ability to reach the target within host cells. When administered in combination with pyrimethamine, a known DHFR inhibitor, the compound produced an additive antiparasitic effect with reduced cytotoxicity toward mammalian cells. For comparison, methotrexate (**59**) displayed stronger inhibition of TcPTR1 (*K*_i_ = 0.11 µM); however, it lacked selectivity and was associated with higher host toxicity (Table 7).

##### Dihydroorotate Dehydrogenase

Dihydroorotate dehydrogenase (DHODH) is a mitochondrial enzyme that catalyzes the oxidation of (*S*)-dihydroorotate to orotate, a key step in the de novo pyrimidine biosynthetic pathway. This route is essential for the synthesis of DNA, RNA, and membrane components, especially in organisms that lack a robust salvage pathway [133]. In *T. cruzi*, DHODH uses fumarate as the terminal electron acceptor, in contrast to the human enzyme, which relies on ubiquinone. These mechanistic and structural differences offer a valuable opportunity for selective inhibitor design. The genetic knockout of TcDHODH leads to parasite death, underscoring its essentiality and validating it as a promising target for Chagas disease chemotherapy [134].

Among the inhibitors developed by Inaoka et al. (2017), compounds (**60**) and (**61**) exhibited the highest potency against TcDHODH [135]. Compound (**60**), substituted with a 6-carboxy-2-naphthyl group at position 5 of the orotate ring, showed an apparent inhibition constant (*K*_iapp_) of 0.024 µM, while (**61**), bearing a 5-carboxy-2-naphthyl moiety, displayed a *K*_iapp_ of 0.033 µM. Both compounds showed exceptional selectivity over the human DHODH, with selectivity indices exceeding 37,000 and 75,000, respectively. These findings highlight the importance of rigid aromatic scaffolds and carboxylic acid functionalities in establishing favorable hydrogen bonding and hydrophobic contacts within the TcDHODH binding site.

Complementarily, de Jesus et al. (2025) applied both 2D and 3D quantitative structure–activity relationship (QSAR) approaches—namely, hologram QSAR (HQSAR) and comparative molecular field analysis (CoMFA)—to a dataset of 64 orotate derivatives [136]. Compounds (**61**) and (**62**) emerged as the most active analogues, with predicted pIC_50_ values of 7.48 and 7.34, corresponding to IC_50_ values of approximately 33 and 46 nM, respectively. These compounds combine extended aromatic systems with short aliphatic linkers, favoring optimal positioning within the active site. The SAR and CoMFA contour analyses revealed that π–π stacking, polar interactions, and steric complementarity are key determinants of binding affinity and selectivity (Table 7).

**Table 7:** Nucleotide metabolism enzymes and lead inhibitors identified for *T. cruzi*.

Compound	Structure	Affinity Data	LogP	Reference
**54**	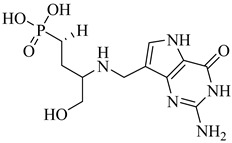	*K*_i_ = 0.6 µM (TcHGXPRT) *Ki* = 0.006 µM (TcHGPRT)	−1.1483	[124]
**55**	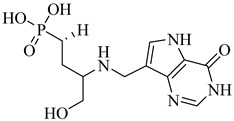	*K*_i_ = 1.6 µM (TcHGXPRT) *K*_i_ = 0.004 µM (TcHGPRT)	−0.7305	[124]
**56**	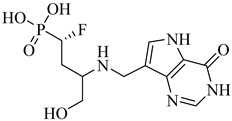	*K*_i_ = 0.7 µM (TcHGXPRT) *K*_i_ = 1.4 µM (TcHGPRT)	−0.4349	[124]
**57**	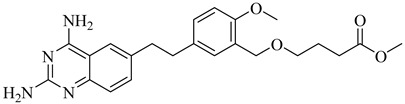	*K*_i_ = 1.3 nM (TcDHFR–TS)	3.0578	[128]
**58**	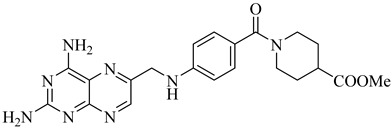	*K*_i_ = 7 µM (TcPTR1)	1.2215	[132]
**59**	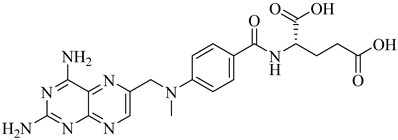	*K*_i_ = 0.11 µM (TcPTR1)	0.2684	[132]
**60**	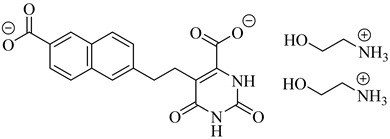	*Ki*_app_ = 0.024 µM (TcDHODH)	−4.8302	[135]
**61**	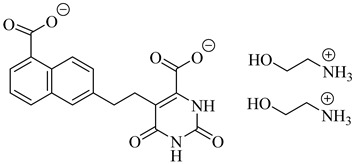	*K*_iapp_ = 0.033 µM IC_50_~33 nM (TcDHODH)	−4.8302	[135,136]
**62**	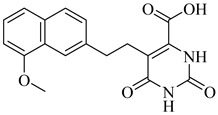	IC_50_~46 nM (TcDHODH)	1.7084	[136]

Abbreviations: TcHGXPRT = *T. cruzi* hypoxanthine–guanine–xanthine phosphoribosyltransferase; TcHGPRT = *T. cruzi* hypoxanthine–guanine phosphoribosyltransferase; TcPTR1 = *T. cruzi* pteridine reductase 1; TcDHFR–TS *= T. cruzi* dihydrofolate reductase–thymidylate synthase; TcDHODH = *T. cruzi* dihydroorotate dehydrogenase; IC_50_ = half-maximal inhibitory concentration; K_i(app)_ = (apparent) inhibition constant; LogP = octanol–water partition coefficient.

### 4.3. Oxidative Stress Defense Mechanisms

#### Metabolism Dependent on Thiol Groups

##### Trypanothione Reductase

In *T. cruzi*, redox homeostasis is sustained by a unique thiol-based system centered on trypanothione, a conjugate of two glutathione molecules and spermidine. The key enzyme in this pathway is trypanothione reductase (TR), a FAD-containing flavoprotein that catalyzes the NADPH-dependent reduction of trypanothione disulfide to its dithiol form [137,138]. This reaction is essential for preserving the parasite’s intracellular reducing environment, thereby enabling the detoxification of reactive oxygen and nitrogen species generated both endogenously and by the host immune system. Unlike mammalian cells, which rely on glutathione and glutathione reductase (GR), *T. cruzi* is entirely dependent on the trypanothione/TR system for oxidative stress management [139].

Due to its strict substrate specificity and absence in the vertebrate host, TR represents a highly selective and chemically tractable target. The inhibition of this enzyme compromises the parasite’s redox balance and viability, particularly during intracellular stages when oxidative stress is intensified [137]. These characteristics position TR as a compelling target for the rational design of novel antitrypanosomal agents.

In 2017, Beltrán-Hortelano et al. published a comprehensive review on inhibitors targeting TR and superoxide dismutase (SOD) from *T. cruzi*, with a focus on identifying promising scaffolds for the treatment of Chagas disease [140]. Among the compounds evaluated, one of the most potent was compound (**63**), a tricyclic derivative that acts as a competitive TR inhibitor, displaying a *K*_i_ of 330 nM and exhibiting no inhibition of human GR. Its compact tricyclic structure is believed to favor optimal positioning within the TR catalytic pocket [141]. Compound (**64**), a hybrid combining diaryl sulfide and BTCP fragments, was identified through the virtual screening of over 100,000 molecules and showed potent TR inhibition with a *K*_i_ of 510 nM, supported by hydrophobic and polar interactions within the active site [142,143]. Compound (**65**), identified as chlorhexidine, also acts as a competitive inhibitor of TR with a *K*_i_ of 2.0 µM, and its selectivity arises from interactions with *T. cruzi*-specific residues Glu19, Trp22, and Met114, which are not conserved in the human GR isoform [144]. Additionally, compound (**66**) (daphnoline), reported in a systematic review and meta-analysis by Mendonça et al. (2018), demonstrated favorable binding in molecular docking studies (DS = −6.247 kcal/mol) through interactions with Thr397 and Glu467 [145]. More importantly, it achieved an 84% parasitological cure rate in a murine model of *T. cruzi* infection, outperforming several other candidates evaluated in vivo [145]. Collectively, these compounds highlight the potential of TR as a validated drug target and provide valuable structural templates for the development of selective and potent antichagasic agents (Table 8).

##### Trypanothione Synthetase

Trypanothione synthetase (TryS) is a multifunctional enzyme that catalyzes the ATP-dependent biosynthesis of trypanothione [*N*^1^,*N*⁸-bis(glutathionyl)spermidine] from two molecules of glutathione and one of spermidine. This unique thiol, exclusive to trypanosomatids, functions as the primary cytosolic redox buffer in these parasites, replacing the GR and thioredoxin reductase systems that are absent in this group [138]. In *T. cruzi*, TryS plays a central role in maintaining redox homeostasis, contributing to the detoxification of ROS and facilitating the parasite’s adaptation to oxidative stress induced by the host immune response and chemotherapeutic agents. The overexpression of TryS enhances parasite proliferation, increases resistance to oxidative stress, and promotes tolerance to nitroheterocyclic drugs [146,147]. Conversely, the pharmacological inhibition of TryS depletes intracellular trypanothione and compromises parasite viability, reinforcing its relevance as a selective therapeutic target.

In this context, Benítez et al. (2016) identified compound (**67**) as one of the most promising *T. cruzi* TryS inhibitors through a targeted screening of 144 structurally diverse compounds [148]. Compound (**67**), a member of the 6-aryl-pyrido [2,3-d]pyrimidine-2,7-diamine class, inhibited 65.4% of TcTryS activity at 30 µM, demonstrating notable selectivity over the homologous enzymes from *T. brucei* (20.9%) and *L. infantum* (13.6%). A more recent review by González-Montero et al. (2024) highlighted (**67**) as the most potent and selective inhibitor of TcTryS reported to date [137]. These findings position (**67**) as a valuable chemical scaffold for the development of more potent and selective TryS inhibitors aimed at targeting redox metabolism in *T. cruzi* (Table 8).

##### Tryparedoxin Peroxidase

In *T. cruzi*, tryparedoxin peroxidases TcCPx (cytosolic) and TcMPx (mitochondrial) play central roles in redox homeostasis. TcCPx operates within a well-characterized cascade involving tryparedoxin I (TcTXNI), trypanothione, and TR, whereas TcMPx can also be reduced in vitro by the same system, although its mitochondrial activity in vivo remains uncertain due to limited evidence of TR localization and mitochondrial NADPH generation [149].

Both isoforms are upregulated during metacyclogenesis and in BZN-resistant strains. TcCPx is additionally secreted under oxidative stress, suggesting functional divergence between isoforms. Beyond peroxide detoxification, TcCPx modulates host immunity by inducing a Th1-type immune response in a peroxidatic cysteine-dependent manner [150]. Proteomic studies have revealed that TcMPx interacts with distinct protein partners under oxidative conditions, supporting context-dependent functions, possibly including chaperone activity or roles in redox signaling [149].

Ferrocenyl diamine derivatives have demonstrated greater trypanocidal activity than BZN, with compound (**68**) exhibiting an IC_50_ of 2.21 µM and compound (**69**) showing comparable potency (IC_50_ = 2.74 µM), both against the Y strain of *T. cruzi* [151]. Notably, TcMPx expression did not increase in strains resistant to these compounds, suggesting that its modulation is not required for adaptation to redox stress under these conditions. This highlights that TcTXNPx, although functionally relevant, is not universally responsive to oxidative challenge and supports its investigation as a component of broader redox adaptation rather than a direct indicator of drug response. Despite the absence of known inhibitors, its essentiality and lack of human homologs continue to support its consideration as a chemically tractable target (Table 8).

**Table 8:** Major enzymes involved in oxidative stress defense in *T. cruzi* and corresponding lead compounds evaluated for inhibitory activity.

Compound	Structure	Affinity Data	LogP	Reference
**63**	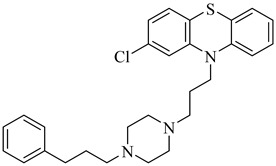	*K*_i_ = 330 nM (TcTR)	6.5832	[141]
**64**	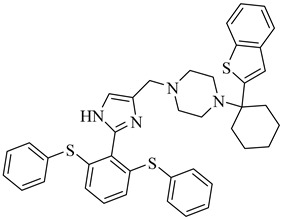	*K*_i_ = 510 nM (TcTR)	10.571	[142,143]
**65**	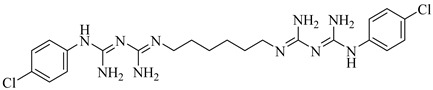	*K*_i_ = 2.0 µM (TcTR)	3.3366	[144]
**66**	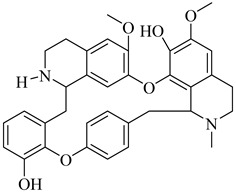	DS = −6.247 kcal/mol (TcTR)	6.2142	[145]
**67**	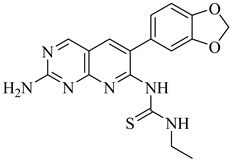	Inhibition (at 30 µM) = 65.4% (TcTryS)	2.309	[148]
**68**	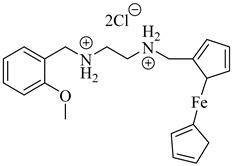	IC_50_ = 2.21 µM (*T. cruzi*)	1.533	[151]
**69**	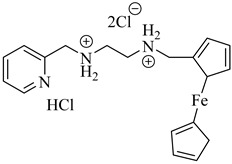	IC_50_ = 2.74 µM (*T. cruzi*)	2.3674	[151]

Abbreviations: DS = docking score; TcTR = *T. cruzi* trypanothione reductase; TcTryS = *T. cruzi* trypanothione synthetase; *K_i_* = inhibition constant; IC_50_ = half-maximal inhibitory concentration; LogP = octanol–water partition coefficient.

### 4.4. Protein Kinases and Signal Transduction

Protein kinases have emerged as compelling molecular targets for the development of new chemotherapeutic agents against *T. cruzi*, owing to their central roles in regulating cell division, differentiation, and survival. The *T. cruzi* kinome comprises approximately 190 predicted protein kinases, corresponding to about 2% of its genome, and includes both conserved enzymes and divergent subfamilies with no close human homologs. This divergence offers opportunities for the development of selective inhibitors with minimal off-target effects in the host [152].

Among the most promising targets is the *T. cruzi* Akt-like kinase (TcAkt), which shares architectural similarities with human Akt isoforms, including a pleckstrin homology (PH) domain and a catalytic kinase domain. Recent biophysical studies—incorporating NMR spectroscopy, molecular dynamics simulations, and docking analyses—have demonstrated that TcAkt adopts an autoinhibited conformation regulated by intramolecular PH–kinase domain interactions, which are disrupted upon binding to phosphatidylinositol phosphate ligands [152]. This conformational change appears essential for activation and may be exploitable for selective inhibition.

Additionally, one parasite-directed compound, (**70**), was shown through molecular docking to engage the catalytic domain of TcAkt, with a binding score of −8.3 kcal/mol [153,154]. Compound (**70**) exhibited potent activity against intracellular *T. cruzi* amastigotes, with an EC_50_ of 1.85 µM, and low cytotoxicity toward human cell lines (LC_50_ > 40 µM), resulting in an SI of 21.6. Initial molecular dynamic simulations suggest binding at the PH domain. Still, more recent AlphaFold-based modeling indicates interaction at the interdomain linker region between the PH and kinase domains, notably involving residues R103, T108, L131, D132, T203, K204, and F435. This interaction is proposed to restrict the flexibility of the interdomain region, preventing the conformational rearrangements required for TcAkt activation. Functional assays revealed that compound (**70**) induces apoptosis-like events in *T. cruzi*, including mitochondrial depolarization, increased ROS levels, and DNA fragmentation.

Another kinase that has undergone functional validation is AGC essential kinase 1 (TcAEK1), a cytosolic serine/threonine kinase involved in cytokinesis, host cell invasion, and intracellular replication. Genetic manipulation via CRISPR/Cas9, combined with chemical inhibition using ATP-analogue-sensitive mutants, has confirmed its essentiality for parasite viability. In *T. cruzi* strains carrying a gatekeeper mutation, the bumped kinase inhibitors (**71**) and (**72**) inhibited epimastigote proliferation with IC_50_ values of 1.55 μM and 2.42 μM, respectively. In metacyclic trypomastigotes, these inhibitors showed increased potency, with IC_50_ values of 421 nM and 578 nM, respectively (Table 9) [155].

In addition to protein kinases, other regulatory components of intracellular signaling networks—such as molecular chaperones—are crucial for sustaining *T. cruzi* viability and facilitating its adaptation to physiological stress. Among these, heat shock proteins (Hsps) represent a conserved family of molecular chaperones involved in stress response and proteostasis.

Among them, Hsp90 is a key cytosolic chaperone with broad functions in maintaining protein quality control, stabilizing essential client proteins, and coordinating cell cycle dynamics and differentiation events. Graefe et al. (2002) demonstrated that the inhibition of Hsp90 using geldanamycin (**73**) at concentrations up to 5 µg/mL elicited a stress response in epimastigotes, marked by the increased expression of secondary chaperones such as Hsp70 and Hsp100, indicating the activation of compensatory proteostasis mechanisms [156]. The same treatment induced G1-phase arrest without direct cytolysis and also impaired morphological transitions between parasite stages, suggesting a role for Hsp90 in the regulation of developmental progression. Together, these findings underline its functional relevance in both homeostasis and environmental adaptation.

Building on these observations, Martínez-Peinado et al. (2021) explored the impact of targeted molecular inhibitors on *T. cruzi* chaperone systems [157]. Among the compounds tested, alvespimycin (**74**) showed pronounced efficacy against epimastigotes, with an IC_50_ of 0.017 µM, leading to reduced proliferation, nuclear disorganization, and blocked differentiation. This was accompanied by increased Hsp70 expression, consistent with Hsp90 inhibition-induced stress signaling. Dorsomorphin (**75**) also inhibited parasite growth, with an IC_50_ of 0.24 µM, though its elevated cytotoxicity in mammalian cells raises concerns about therapeutic applicability. These data support the candidacy of Hsp90/Hsp83 as a validated and promising molecular target for anti-*T. cruzi* drug development.

**Table 9:** Protein kinase targets in *T. cruzi* and their inhibitors.

Compound	Structure	Affinity Data	LogP	Reference
**70**	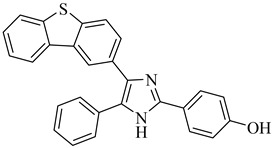	EC_50_ = 1.85 µM (*T. cruzi*—amastigote) Binding Score = −8.3 kcal/mol (TcAkt)	7.4842	[153,154]
**71**	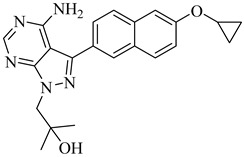	IC_50_ = 1.55 μM (*T. cruzi*—epimastigote) IC_50_ = 421 nM (*T. cruzi*—trypomastigote)	3.5408	[155]
**72**	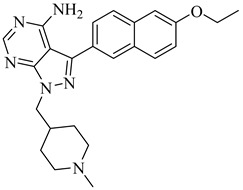	IC_50_ = 2.42 μM (*T. cruzi*—epimastigote) IC_50_ = 578 nM (*T. cruzi*—trypomastigote)	3.9692	[155]
**73**	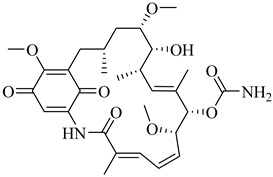	—	2.4084	[156]
**74**	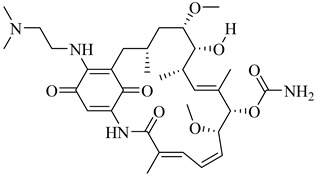	IC_50_ = 0.017 μM (*T. cruzi*—epimastigote)	1.9132	[157]
**75**	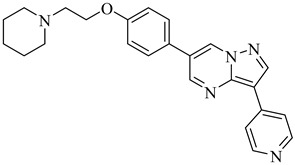	IC_50_ = 0.24 μM (*T. cruzi*—epimastigote)	4.323	[157]

Abbreviations: IC_50_ = half-maximal inhibitory concentration; EC_50_ = half-maximal effective concentration; LogP = octanol–water partition coefficient.

### 4.5. DNA Replication and Epigenetic Regulation

#### 4.5.1. DNA Topoisomerases

DNA topoisomerases are increasingly recognized as valuable targets in the development of new therapeutic strategies against *T. cruzi*. These enzymes are involved in fundamental processes such as DNA replication, recombination, and the cellular response to genomic stress. In particular, topoisomerase 3α (Topo3α) has been shown to contribute significantly to genome maintenance. Functional studies involving gene disruption have demonstrated that the absence of *TcTopo3α* impairs amastigote proliferation, increases cellular dormancy, and leads to a heightened sensitivity to genotoxic agents, underscoring its role in homologous recombination repair and the replication stress response [158].

While several clinically approved topoisomerase inhibitors have been evaluated for antitrypanosomal activity, most have shown limited efficacy against *T. cruzi*. Among them, idarubicin (**76**) stood out by exhibiting measurable trypanocidal activity against both Sylvio X10 and Esmeraldo strains, with half-maximal growth inhibitory concentration (GI_50_) values of 38.5 µM and 24.6 µM, respectively [159]. Although these micromolar-range potencies may limit its direct repurposing, the observed activity reinforces the potential of anthracycline scaffolds as starting points for the development of selective topoisomerase-targeting agents against *T. cruzi*.

More recently, a novel class of cyanotriazole-based compounds has emerged as a promising chemotype targeting topoisomerase IIα in trypanosomatids [160]. The lead compound, (**77**), exhibited potent antiparasitic activity, with an EC_50_ of 78 nM, and displayed low cytotoxicity toward mammalian cells, with a half-maximal cytotoxic concentration (CC_50_) exceeding 50 µM, indicating a favorable selectivity window. Notably, it achieved sterile cure in a chronic *T. cruzi* infection model. Structural analyses revealed covalent binding to cysteine 477, a residue conserved in the parasite enzyme and absent in the human orthologue, thereby providing a strong rationale for selective inhibition. Two structurally related analogues, (**78**) and (**79**), also demonstrated nanomolar activity against *T. cruzi* (EC_50_ values of 69 nM and 87 nM, respectively) along with high selectivity indices. Nonetheless, only compound (**77**) progressed to in vivo studies due to its superior pharmacokinetic profile, including oral bioavailability and central nervous system penetration (Table 10).

#### 4.5.2. Bromodomain Proteins

##### Bromodomain Factor 3

Bromodomains are protein modules involved in epigenetic regulation that interact with acetylated lysine residues and modulate gene expression. In *T. cruzi*, at least eight bromodomain-containing proteins (TcBDF1–TcBDF8) have been identified, each playing distinct roles in the parasite’s biology. While some of these proteins are involved in transcriptional regulation, others contribute to structural processes and cellular differentiation, making them attractive targets for therapeutic intervention [161].

Among these factors, bromodomain factor 3 (TcBDF3) exhibits unique structural characteristics compared to human BET bromodomains, which contain two bromodomains and an Extra-Terminal (ET) domain. In contrast, TcBDF3 possesses only a single bromodomain linked to an ET domain, suggesting a distinct structural framework that could enable selective pharmacological targeting. Additionally, recent studies indicate that TcBDF3 is crucial for parasite differentiation and displays an atypical subcellular distribution, localizing in the cytoplasm, flagellum, and flagellar pocket. This distribution suggests potential interactions with cytoskeletal components and mechanisms involved in parasite adaptation to the intracellular environment [162].

TcBDF3 overexpression affects the parasite’s life cycle progression and infectivity. Its association with acetylated tubulin in the flagellum implies a role in regulating *T. cruzi* morphology and motility, which are essential for virulence. Structural studies have revealed that its acetylated ligand-binding site exhibits distinct hydrophobic properties, highlighting the potential of bromodomain inhibition as a promising approach for the development of novel antiparasitic therapies [163].

In 2024, Alonso and colleagues advanced the identification of selective *TcBDF3* inhibitors, focusing on small molecules capable of modulating its interaction with acetylated proteins. Biophysical and computational analyses demonstrated that inhibiting this bromodomain disrupts essential functions in *T. cruzi* [164]. Among the tested compounds, 1,3,4-oxadiazoles exhibited high affinity for *TcBDF3*, with *K*_d_ values ranging from 1.7 to 4.8 µM in thermal shift assays (DSF) and IC_50_ values between 2.4 and 10.5 µM in fluorescence polarization (FP) assays. Compound (**80**) displayed the highest affinity (*K*_d_ = 1.7 µM, IC_50_ = 2.4 µM), followed by its cyclic derivatives (**81**) (*K*_d_ = 4.0 µM, IC_50_ = 8.4 µM) and (**82**) (*K*_d_ = 4.8 µM, IC_50_ = 10.5 µM). These inhibitors effectively block the hydrophobic pocket of the bromodomain, preventing interactions with acetylated substrates and impairing parasite viability. Furthermore, they demonstrated significant trypanocidal activity, with IC_50_ values below 10 µM against trypomastigotes and amastigotes. Notably, (**81**) exhibited the highest potency against the infectious form of the parasite (IC_50_ = 1.67 µM for trypomastigotes and 6.9 µM for amastigotes) (Table 10).

**Table 10:** Summary of recent therapeutic approaches targeting DNA replication and epigenetic regulation mechanisms in *T. cruzi*.

Compound	Structure	Affinity Data	LogP	Reference
**76**	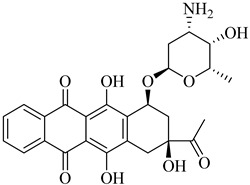	GI_50_ = 38.5 µM (Sylvio X10 strain) GI_50_ = 24.6 µM (Esmeraldo strain)	1.0203	[159]
**77**	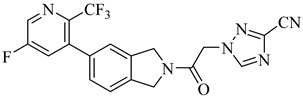	EC_50_ = 78 nM (Topo IIα)	2.91208	[160]
**78**	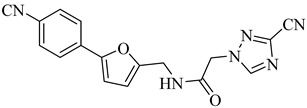	EC_50_ = 69 nM (Topo IIα)	2.27697	[160]
**79**	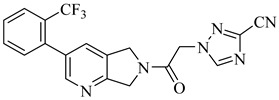	EC_50_ = 87 nM (Topo IIα)	2.77298	[160]
**80**	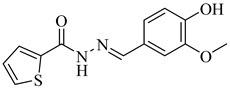	IC_50_ = 2.4 µM *K*_d_ = 1.7 µM (TcBDF3)	2.2262	[164]
**81**	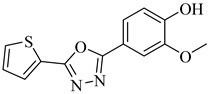	IC_50_ = 8.4 µM *K*_d_ = 4.0 µM (TcBDF3)	3.1793	[164]
**82**	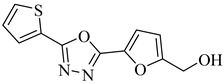	IC_50_ = 10.5 µM *K*_d_ = 4.8 µM (TcBDF3)	2.5504	[164]

Abbreviations: GI_50_ = half-maximal growth inhibitory concentration; TcTopoIIα = *T. cruzi* DNA topoisomerase II alpha; TcBDF3 = *T. cruzi* bromodomain factor 3; EC_50_ = half-maximal effective concentration; *K_d_* = dissociation constant; LogP = octanol–water partition coefficient.

### 4.6. Host-Parasite Interaction and Immune Evasion

#### 4.6.1. Transfer of Sialic Acid

##### Trans-Sialidase

*T. cruzi* trans-sialidase (TcTS) belongs to the glycoside hydrolase family 33 (GH-33) and plays a crucial role in the parasite’s survival. Unlike classical neuraminidases, TcTS transfers sialic acid residues from host glycoconjugates to the surface of *T. cruzi*, protecting it from the immune response and facilitating cell invasion. Structurally, the enzyme features an *N*-terminal catalytic domain, where conserved residues such as Arg35, Arg245, Arg314, Tyr342, Glu230, and Asp59 are essential for its activity, and a *C*-terminal SAPA (shed acute-phase antigen) domain, which prolongs its stability in plasma and enhances its role in infection [165].

In addition to promoting parasite adhesion and invasion, TcTS significantly modulates the host immune system. The enzyme inhibits T lymphocyte activation, induces the apoptosis of immune cells, and interferes with complement system activity, contributing to immune evasion. During the chronic phase of Chagas disease, the continuous activity of TcTS is associated with immune dysfunction and the progressive development of cardiac and digestive damage. Its biological relevance and the absence of catalytically identical homologs in humans make it a strategic target for the development of selective inhibitors, although specificity must be ensured to avoid interactions with other human sialidases [166,167].

Neres et al. (2009) conducted a virtual screening and synthesized 23 benzothiazole derivatives, evaluating their ability to inhibit TcTS through in vitro enzymatic assays [168]. Compound (**83**) was identified as the most promising, exhibiting 92% inhibition of TcTS at 1 mM, with an IC_50_ of 0.15 mM in the MuNANA assay (4-methylumbelliferyl-*N*-acetylneuraminic acid assay) and 0.29 mM in the sialic acid transfer assay. Kinetic studies indicated a mixed or non-competitive inhibition mechanism. In 2018, Kashif et al. synthesized phthalimide derivatives of 3-amino-3-arylpropanoate and identified potential TcTS inhibitors through molecular docking followed by in vitro enzymatic assays [169]. Compounds (**84**) and (**85**) exhibited the enzymatic inhibition of TcTS at 82.5% and 76.5% at 1 mM, respectively. The most promising compound was (**86**), which demonstrated a computational affinity of −11.1 kcal/mol and an enzymatic inhibition of 86.9%. Additionally, (**86**) showed significant trypanocidal activity, with LC_50_ values of 52.70 µM (NINOA) and 46.19 µM (INC-5), indicating its therapeutic potential. Also in 2018, Chen et al. synthesized aryl α-aminophosphonate derivatives and evaluated their TcTS inhibition using the MuNANA fluorimetric assay. Compounds (**87**) and (**88**) were identified as non-competitive inhibitors, with IC_50_ values of 0.23 mM and 0.21 mM, respectively, and *K*_i_ values of 190 and 140 µM. The study by Vázquez-Jiménez et al. (2021) highlighted compound (**89**), a benzoic acid derivative, which exhibited strong TcTS inhibition (87.6%) and computational affinity (−9.6 kcal/mol) [170]. However, its trypanocidal activity was limited, with low cell lysis in *T. cruzi* trypomastigotes from the NINOA (0%) and INC-5 (22.91%) strains, possibly due to cell permeability issues (Table 11). Therefore, inhibiting this enzyme could reduce parasite burden, hinder *T. cruzi* immune evasion, and prevent disease progression. However, challenges such as selectivity, bioavailability, and metabolic stability of inhibitors still need to be overcome to enable the development of effective compounds against this enzyme.

#### 4.6.2. Proteases (Cellular Invasion and Immune Escape)

##### Cysteine Protease (Cruzain)

Cruzain is the main protease of *T. cruzi* and a promising target for the development of new drugs against Chagas disease. Its distribution across different parasite stages suggests that it plays essential roles in amino acid acquisition and immune evasion. Structurally, cruzain has a catalytic site composed of Cys25, His162, and Asp182, which facilitate its proteolytic activity. The substrate-binding site contains both hydrophobic and hydrophilic regions that influence its selectivity, making it a key focus in inhibitor design [171,172].

Cruzain inhibitors can be classified as peptidomimetics or non-peptidic, as well as reversible or irreversible. Many of these inhibitors contain electrophilic groups (warheads), such as α,β-unsaturated carbonyls and nitriles, which covalently interact with the catalytic cysteine. Hybrid strategies, such as combining isatins with thiosemicarbazones, have led to the development of more potent and selective compounds. Although several inhibitor classes have been studied, challenges related to selectivity and stability still limit their therapeutic applicability [173,174,175].

In recent years, combinatorial chemistry and bioisosterism have emerged as innovative approaches to optimizing the efficacy and safety of cruzain inhibitors. The replacement of problematic functional groups, such as amides with thiazoles, has been explored to enhance selectivity and reduce toxicity [176]. The integration of molecular modeling and pharmacokinetic studies has been crucial in accelerating the discovery of new drug candidates [177,178]. Thus, the search for more effective cruzain inhibitors remains a promising strategy for Chagas disease treatment.

Prates et al. (2024) reviewed and analyzed recent advances in the discovery of new cysteine protease cruzain inhibitors, a key enzyme in the life cycle of *T. cruzi* and a promising target for Chagas disease treatment [179]. The study identified promising compounds, assessed their efficacy through enzymatic assays and molecular docking, and discussed their medicinal chemistry perspectives, focusing on drug optimization. Among peptidomimetic inhibitors, compounds (**90**), (**91**), and (**92**) [180] demonstrated nanomolar enzymatic activity, with IC_50_ values of 1.3 nM (0.0013 µM), 0.081 nM (0.000081 µM), and 0.09 nM (0.00009 µM), respectively. Among non-peptidomimetic inhibitors, compounds (**93**) [181], (**94**), and (**95**) [182] stood out due to their potency and selectivity, with IC_50_ values of 120 nM (0.12 µM), 40 nM (0.04 µM), and 10 nM (0.01 µM), respectively (Table 12).

### 4.7. Drug Targets and Resistance Mechanisms

#### Nitroreductases

##### Nitroreductase Type I

Type I nitroreductase (TcNTR) from *T. cruzi* plays a key role in activating BZN and NFX. This enzyme catalyzes the bi-electronic reduction of the nitro group in these drugs, generating toxic metabolites that contribute to parasite death. TcNTR is an FMN-dependent flavoprotein (flavin mononucleotide) that uses NADH as an electron donor to reduce nitroaromatic compounds [183]. Structural studies indicate that this enzyme has a unique 23-residue insertion (199–222), which may be involved in membrane interactions, particularly in cardiolipin-rich regions [184]. This structural feature likely influences TcNTR’s cellular localization and substrate interactions.

TcNTR is classified as a type I nitroreductase, catalyzing the bi-electronic reduction of nitrocompounds, including NFX [185]. NFX activation produces a highly cytotoxic unsaturated open-chain nitrile metabolite, which affects both parasite and host cells. Initially, this effect was thought to be mediated by ROS. However, recent studies show that TcNTR directly reduces NFX to cytotoxic metabolites with minimal ROS involvement [186]. This selectivity is due to TcNTR’s limited expression in human cells, making NFX more selective for *T. cruzi*.

BZN resistance has been linked to deletions and mutations in the TcNTR gene, reducing drug bioactivation [185]. However, Mejía-Jaramillo et al. (2011) [186] found that resistant clones can retain functional TcNTR, suggesting additional resistance mechanisms. These include changes in energy metabolism that mitigate the toxic effects of BZN-derived metabolites, the overexpression of oxidative stress response proteins such as tryparedoxins and SOD, and the modulation of membrane transporters that reduce drug uptake or increase efflux [184]. Thus, BZN resistance likely involves multiple adaptive pathways beyond TcNTR mutations, highlighting the need for new therapeutic targets or combination strategies for Chagas disease treatment.

Menozzi et al. (2023) evaluated a series of 3-nitrotriazole, 2-nitroimidazole, and triazole derivatives, identifying compound (**96**) as the most potent and selective candidate against *T. cruzi*, with an IC_50_ of 0.39 µM and an SI of 3077 [187]. This compound exhibited a metabolic rate up to threefold higher than BZN when incubated with TcNTR-I, suggesting that its mode of action is primarily driven by NTR-mediated bioactivation. Carvalho et al. (2023) synthesized 46 2-nitroimidazole derivatives and demonstrated that compound (**97**) was the most efficiently metabolized by TcNTR with an observed rate constant (*K*_obs_) of 1.27 s⁻^1^, exceeding that of BZN (*K*_obs_ = 0.30 s⁻^1^) [188]. Compounds (**98**) and (**99**) also displayed favorable kinetic profiles, supporting their activation through TcNTR and the subsequent generation of parasite-selective toxic species. Similarly, Pitombeira et al. (2024) designed hybrid molecules, integrating the 2-nitroimidazole scaffold with *N*-acylhydrazone motifs [189]. Among them, compound (**100**), bearing a meta-hydroxyl substituent, exhibited an IC_50_ of 5.4 µM (SI = 119.5) and *K*_obs_ values of 0.32 and 0.47 at 25 and 50 µM, respectively (comparable to BZN) (Table 13). Additional meta-substituted derivatives, such as methoxy-, chloro-, and fluoro-substituted analogues, also demonstrated enhanced TcNTR-I activation. Collectively, these findings reinforce the relevance of NTR-mediated activation in the antiparasitic activity of nitroaromatic compounds and highlight promising scaffolds for the development of new treatments for Chagas disease.

Although the following sections detail traditional inhibitors and drug targets in *T. cruzi*, many of these targets can, in principle, be adapted for PROTAC-based degradation depending on the availability of selective ligands and the accessibility of degron motifs. These targets serve as a foundation for evaluating the feasibility of TrypPROTAC design, as discussed in Section 6.

## 5. PROTACs: Mechanism of Action, Therapeutic Applications, and Key Challenges

**Figure 4:**
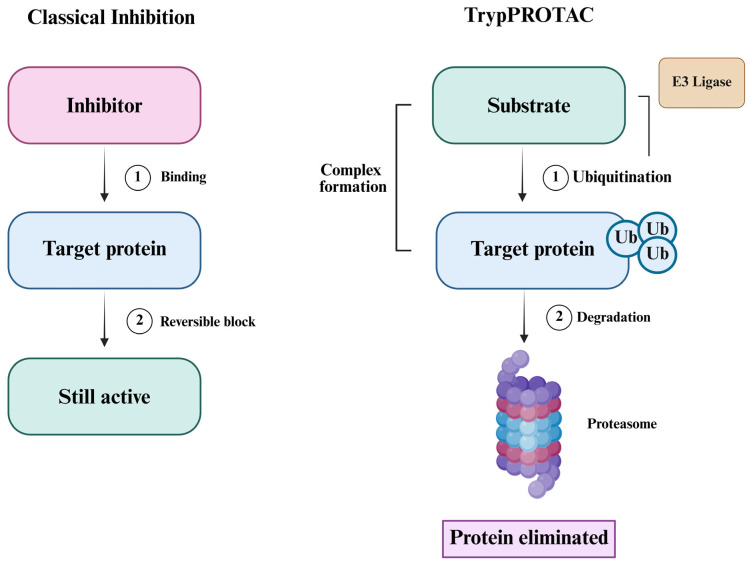
Comparison between classical inhibition and TrypPROTAC-mediated protein degradation. Created in BioRender. Dos santos, J. (2025) https://biorender.com/r22f256, accessed on 18 May 2025.

The strategy of TPD has transformed drug discovery by enabling the selective elimination of disease-associated intracellular proteins [190]. Unlike conventional inhibitors that modulate protein activity by binding to functional sites, PROTACs induce the irreversible degradation of target proteins via the ubiquitin–proteasome system (UPS). This strategy significantly expands the druggable proteome, targeting previously undruggable proteins, such as transcription factors and scaffold proteins [191]. Moreover, PROTACs offer a novel approach to overcoming therapeutic resistance, providing more durable and effective modulation of disease-relevant proteins (Figure 4) [192,193].

### 5.1. Mechanism of Action

PROTACs are bifunctional molecules designed to induce the selective degradation of intracellular proteins via the UPS [194]. They consist of the following three essential domains: a ligand for the protein of interest (POI), a ligand for an E3 ubiquitin ligase, and a chemical linker. The linker’s composition, length, and flexibility are crucial for stabilizing the ternary complex (POI–PROTAC–E3 ligase), directly influencing degradation efficiency [195,196].

Once the ternary complex is formed, the PROTAC recruits the E3 ligase, triggering the transfer of multiple ubiquitin molecules to specific residues of the POI. This post-translational modification marks the protein for proteolysis by the 26S proteasome, where it is unfolded, translocated, and cleaved into small peptides. Unlike traditional inhibitors, which merely modulate protein function, PROTACs completely remove the target protein from the cellular environment, offering a unique, orthogonal mechanism of action that mitigates resistance due to mutations or overexpression of the target [196,197,198].

### 5.2. Therapeutic Applications of PROTACs

PROTACs have become a groundbreaking tool in drug discovery, enabling the selective degradation of disease-relevant proteins through hijacking the UPS. Though primarily explored in oncology, their potential has expanded into the treatment of infectious diseases, including bacterial and viral infections, highlighting their versatility [199,200,201].

#### 5.2.1. PROTACs in Oncology: Targeting Oncogenic Proteins

In oncology, PROTACs have shown considerable promise by facilitating the selective elimination of oncogenic drivers involved in transcriptional regulation, cell cycle control, and receptor tyrosine kinase (RTK) signaling [202,203,204]. Unlike classical inhibitors, which require sustained binding to active sites, PROTACs function catalytically and transiently. This catalytic mode of action promotes degradation via the UPS while also reducing the likelihood of resistance arising from target overexpression or mutational escape [204,205,206].

A notable example is the degradation of bromodomain-containing protein 4 (BRD4), a coactivator regulating myelocytomatosis oncogene (MYC) transcription. Various PROTACs have demonstrated the ability to induce potent BRD4 degradation, leading to sustained MYC suppression and significant antitumor activity in hematological and solid tumor models [203,207,208]. Another relevant application involves the targeted degradation of cyclin-dependent kinases 4 and 6 (CDK4/6) in hormone receptor-negative breast cancer, offering an alternative to conventional kinase inhibition strategies [204].

PROTACs have also been explored for targeting RTKs such as the epidermal growth factor receptor (EGFR) and human epidermal growth factor receptor 2 (HER2), which are commonly altered in solid tumors. Their ability to mediate RTK degradation not only abrogates downstream signaling; it also prevents receptor recycling and compensatory reactivation, which are challenges frequently observed with reversible kinase inhibitors. By inducing RTK degradation, PROTACs help overcome acquired resistance in cancer therapies, enhancing the efficacy of treatments in resistant tumors [202,209].

#### 5.2.2. PROTACs for Antibacterial Drug Discovery

Classical PROTACs are not directly applicable to bacteria due to the absence of the UPS [210,211]. To address this, BacPROTACs have been developed as bifunctional molecules that exploit bacterial proteolytic machineries, notably the ClpC-ClpP complex (caseinolytic protease system), to induce the targeted degradation of essential bacterial proteins [210,212]. This event-driven mechanism allows repeated engagement of the target and does not depend on enzyme inhibition, thereby broadening the chemical space of tractable targets.

Structural features such as disordered termini or degron-like motifs are critical for recognition by bacterial unfoldases and impact degradation efficiency [210,212]. Beyond direct bactericidal activity, BacPROTACs can enhance the efficacy of existing antibiotics, particularly against tolerant or persistent populations [212]. Their modularity enables the rapid reprogramming of target specificity, rendering them valuable both as antimicrobial agents and as probes for chemical biology and target validation in bacterial systems [213].

#### 5.2.3. PROTACs in Antiviral Therapies

In the context of antiviral research, TPD has also been explored as a strategy to modulate host or viral proteins that are difficult to inhibit using classical approaches. By recruiting E3 ligases, PROTACs promote the selective degradation of key factors involved in viral replication, latency, or immune evasion, often with prolonged pharmacodynamic effects due to their catalytic mode of action [214,215].

Notably, certain viruses such as Human Immunodeficiency Virus type 1 (HIV-1) and Human Papillomavirus (HPV) manipulate host degradation pathways to subvert antiviral responses. Redirecting E3 ligases to degrade viral proteins can potentially restore host immunity and impair viral persistence, even in drug-resistant contexts [216,217]. Moreover, PROTACs targeting enzymes or structural components of respiratory viruses, including influenza and coronaviruses, have shown preclinical promise in disrupting various stages of the viral life cycle [218,219].

Despite these advances, challenges remain. These include the limited availability of high-affinity ligands for viral targets, suboptimal membrane permeability due to the large molecular size of PROTACs, and the need for sustained intracellular exposure to achieve effective degradation [215,220]. Nevertheless, PROTACs offer a mechanistically distinct approach for antiviral therapy with potential to address limitations of current treatments.

### 5.3. Advantages and Challenges of PROTACs in Drug Development

PROTACs represent a significant advancement in drug development, offering a unique alternative to conventional small-molecule inhibitors. Unlike traditional inhibitors, which block protein activity, PROTACs promote the complete elimination of the target protein. This reduces the likelihood of acquired resistance, a common challenge in therapies that rely on active-site occupancy [221]. Moreover, PROTACs function catalytically, enabling sustained protein degradation at lower doses, which could reduce toxicities associated with prolonged drug exposure [222].

PROTAC technology opens new possibilities in drug discovery by enabling the degradation of previously “undruggable” targets, such as transcription factors and scaffolding proteins. By engaging the UPS, PROTACs modulate proteins that lack conventional druggable binding sites [223,224].

A key strength of PROTACs is their enhanced selectivity. Choosing the right E3 ligases allows for the targeting of specific protein isoforms or mutant variants, improving therapeutic precision. This capability, along with the spatial regulation of protein degradation, increases the precision and efficacy of treatment [223,225,226].

Despite these advantages, challenges remain. The stability of the ternary complex (PROTAC, target protein, and E3 ligase) is highly dependent on dynamic interactions, complicating rational design [224]. Moreover, while the repertoire of E3 ligases is expanding, the limited number of well-characterized ligases still restricts PROTAC applications [227].

From a pharmacokinetic and pharmacodynamic perspective, PROTACs face challenges related to their high molecular weight and polarity, which can hinder bioavailability and cellular permeability. These properties may limit their effectiveness in certain therapeutic areas, particularly where tissue penetration is crucial [222].

Additionally, off-target toxicity remains a concern due to the potential degradation of essential proteins in sensitive tissues or organs. There is also overlap between PROTACs and “molecular glues,” which also utilize the UPS for protein degradation, making it difficult to optimize these molecules for specific applications [228].

## 6. Molecular Design of TrypPROTACs for *T. cruzi*

**Figure 5:**
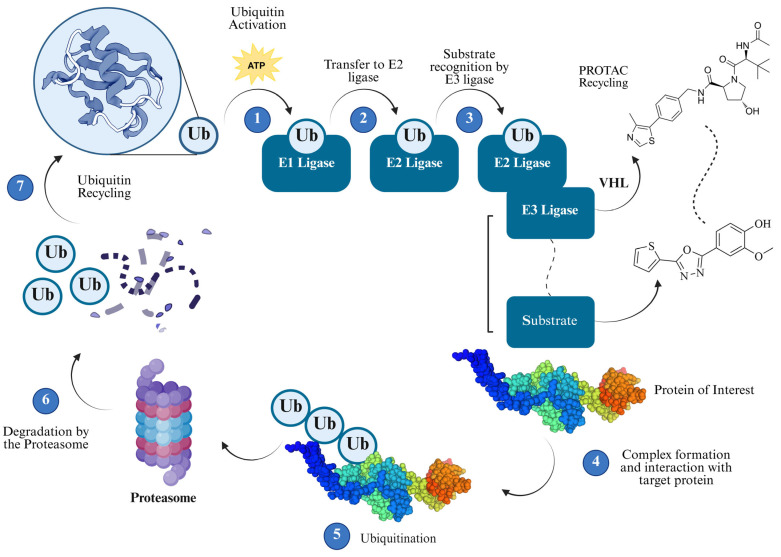
Mechanism of TrypPROTAC-mediated degradation of parasite proteins in *T. cruzi*. Created in BioRender. Dos santos, J. (2025) https://biorender.com/r22f256, accessed on 18 May 2025.

The rational design of TrypPROTACs involves combining parasite-specific target biology with ligand chemistry to facilitate selective protein degradation in *T. cruzi*. Key steps include identifying essential parasite proteins, selecting high-affinity ligands, and ensuring compatibility with linker attachment and E3 ligase recruitment. By integrating these components, PROTAC molecules can be constructed to induce the selective and validated degradation of critical proteins within the parasite (Figure 5) [201,229].

### 6.1. Prioritized Targets and Representative Ligands

Given the absence of validated TrypPROTACs or experimental reports on targeted protein degradation in kinetoplastids, we proposed a rational framework to identify suitable molecular targets for PROTAC design. Target selection was guided by biological essentiality, known ligandability, subcellular localization, and structural features compatible with ternary complex formation. Priority was given to intracellular proteins that are divergent from human homologues and are critical for parasite survival or infectivity. Notably, TcCYP51, TcTR, TcAkt, Hsp90, topoisomerase IIα (TcTopoIIα), TcBDF3, and cruzain were highlighted as the most promising candidates.

To support this prioritization, our analysis extended beyond druggability alone, incorporating parameters directly relevant to targeted protein degradation strategies. All selected targets have been shown to be essential for *T. cruzi* viability through genetic, biochemical, or pharmacological studies. Moreover, they remain functionally active across distinct life cycle stages of the parasite, including replicative epimastigotes and intracellular amastigotes, reinforcing their relevance for interventions targeting both acute and chronic phases of infection. TcTR and Hsp90, for example, are involved in redox balance and cellular stress responses, respectively, while TcBDF3 and TcAkt contribute to differentiation, proliferation, and intracellular signaling.

Resistance mechanisms have been reported in association with traditional inhibitors of TcCYP51 and cruzain, particularly under prolonged chemotherapeutic pressure [230,231]. These findings suggest that degradation-based approaches may provide an alternative route to circumvent adaptive resistance. From a cellular and structural perspective, the majority of these targets are cytosolic or nuclear, positioning them within the reach of the UPS and, thus, amenable to degradation via PROTACs. Hsp90, in particular, represents a compelling candidate given its prior successful degradation by PROTACs in human cells, offering a translational precedent for its application in trypanosomatids [232]. Altogether, the integration of functional essentiality, subcellular accessibility, and ligand compatibility provides a robust foundation for prioritizing these targets in the rational development of TrypPROTACs.

Following target selection, representative ligands were chosen based on their antiparasitic potency and physicochemical properties. Special attention was given to their partition coefficient (LogP) to ensure favorable cell permeability while minimizing aggregation potential and off-target effects (Table 14) [233].

For TcCYP51, representative ligands include compound (**31**) (*K*_d_ = 8.4 nM) and compound (**32**) (*K*_d_ < 10.0 µM) [44]. TcCYP51 is involved in ergosterol biosynthesis, which is essential for parasite membrane integrity. While CYP51 is a well-validated target, the LogP values of compound (**31**) (LogP = 6.85) and compound (**32**) (LogP = 5.28) are markedly elevated, posing challenges for PROTAC development due to potential issues with solubility and passive diffusion. Nonetheless, TcCYP51 remains a valuable target, particularly when approached with tailored linker strategies or solubility-enhancing modifications.

TcTR is another essential target for PROTACs, with ligands such as compound (**63**) (*Ki* = 330 nM) and compound (**64**) (*Ki* = 510 nM) [141,142]. TcTR plays a critical role in maintaining redox homeostasis in *T. cruzi* and lacks a functional homolog in humans, reinforcing its potential for selective therapeutic intervention. Although TcTR is a soluble enzyme, its compact globular architecture may limit the formation of productive ternary complexes. Moreover, the high lipophilicity of its ligands, especially compound (**64**) (LogP = 10.57), may hinder aqueous solubility and increase nonspecific binding. Despite these constraints, TcTR remains a compelling candidate for PROTAC development when coupled with the appropriate linker design and physicochemical optimization.

For TcAkt, compound (**71**) is a representative ligand, exhibiting IC_50_ values of 1.55 μM against epimastigotes and 421 nM against trypomastigotes of *T. cruzi* [155]. TcAkt plays a pivotal role in parasite survival and differentiation and displays notable structural divergence from its human ortholog, favoring selective inhibition. The LogP of compound (**71**) is 3.54, which falls within the optimal range for passive cell permeability and supports its suitability for incorporation into PROTAC constructs. Given its central role in signaling pathways, TcAkt emerges as a high-priority target for TPD.

For Hsp90/Hsp83, compound (**74**) is highlighted as a representative ligand, exhibiting potent antiparasitic activity in *T. cruzi* epimastigotes with an IC_50_ of 0.017 µM [157]. This compound targets the conserved *N*-terminal ATP-binding pocket of Hsp90, a cytosolic molecular chaperone essential for protein folding, cell cycle progression, and parasite differentiation. The LogP of (**74**) falls within the ideal range for passive diffusion, and its physicochemical properties are compatible with linker functionalization. Notably, the compound has already been utilized as a warhead in PROTACs designed to degrade human Hsp90, underscoring its suitability for adaptation in TrypPROTAC constructs. The essentiality of Hsp90/Hsp83 and the availability of high-affinity ligands strengthen its candidacy for targeted protein degradation in kinetoplastids.

Compounds (**77**) and (**78**) are representative ligands for TcTopoIIα, exhibiting potent inhibitory activity with IC_50_ values of 78 nM (LogP = 2.91) and 69 nM (LogP = 2.27), respectively [160]. Both compounds show favorable lipophilicity, supporting efficient cellular uptake. Compound (**77**) is particularly notable for its covalent interaction with Cys477, potentially enabling more sustained degradation of the target. In contrast, compound (**78**), though non-covalent, retains high potency. TcTopoIIα is functionally indispensable, with essential roles in DNA replication, recombination, and stress response, and it presents a structurally accessible site for PROTAC engagement.

Representative ligands targeting TcBDF3 include compound (**80**) (IC_50_ = 2.4 µM, *K*_d_ = 1.7 µM, Log P = 2.23) and compound (**81**) (IC_50_ = 8.4 µM, *K*_d_ = 4.0 µM, Log P = 3.27) [166]. These ligands exhibit moderate affinity and LogP values within or near the ideal range for passive membrane diffusion, making them promising for PROTAC-based applications. TcBDF3, a bromodomain-containing factor, is crucial for parasite differentiation, morphology, and infectivity, and it is structurally divergent from its mammalian counterparts. Its hydrophobic binding pocket is particularly amenable to linker conjugation, enhancing its attractiveness as a PROTAC target.

For cruzain, the major cysteine protease of *T. cruzi*, representative ligands include compound (**91**) (IC_50_ = 0.081 nM, LogP = 5.04) and compound (**95**) (IC_50_ = 0.01 µM, LogP = 5.19) [180,182]. These ligands demonstrate exceptional potency and reinforce the therapeutic relevance of this target. However, their relatively high lipophilicity may lead to issues related to solubility, aggregation, and nonspecific interactions, which are factors that should be carefully addressed in PROTAC design. Despite these challenges, the biological significance of cruzain and the availability of potent, well-characterized ligands support its inclusion as a candidate for targeted degradation, particularly when coupled with strategies that mitigate its physicochemical liabilities.

Together, these prioritized targets and their representative ligands provide a robust and mechanistically diverse foundation for the rational development of TrypPROTACs against *T. cruzi*, integrating biological essentiality with tractable structural and pharmacological profiles.

**Table 14:** Prioritized molecular targets and representative ligands for PROTAC development in *T. cruzi*.

Target	Ligand	Affinity Data	LogP	Target Rationale	Comment
TcCYP51	(**31**)	*K*_d_ = 8.4 nM	6.85	Essential for ergosterol biosynthesis and membrane integrity. Active across life stages. Resistance to azoles reported. Intracellular and structurally accessible.	Potent but highly lipophilic ligands; elevated LogP may impair solubility and passive diffusion. Requires optimized linker and solubility-enhancing strategies.
	(**32**)	*K*_d_ < 10.0 nM	5.28		
TcTR	(**63**)	*K*_i_ = 330 nM	6.58	Key redox enzyme with no human homolog. Essential under oxidative stress. Cytosolic and suitable for UPS access.	Compact soluble target; high ligand lipophilicity and globular shape may limit ternary complex formation. Demands polarity-balanced linker design.
	(**64**)	*K*_i_ = 510 nM	10.57		
TcAkt	(**71**)	IC_50_ = 421 nM	3.54	Involved in signaling, proliferation, and differentiation. Structurally divergent. Cytoplasmic and PROTAC-compatible despite no current precedents.	Favorable LogP and cellular accessibility; minimal structural modification required for PROTAC conversion.
Hsp90	(**74**)	IC_50_ = 0.017 µM	1.91	Essential chaperone for stress response and differentiation. Cytosolic, ligand-accessible, and successfully degraded by PROTACs in human cells.	Known PROTAC warhead; high-affinity ligand with optimal LogP and established use in mammalian systems.
TcTopoIIα	(**77**)	IC_50_ = 78 nM	2.91	Crucial for DNA replication and repair. Nuclear, with high turnover and covalent ligands supporting sustained degradation.	Potent ligands with ideal LogP. Covalent binding (Cys477) may enhance degradation efficiency.
	(**78**)	IC_50_ = 78 nM	2.27		
TcBDF3	(**80**)	IC_50_ = 2.4 µM *K*_d_ = 1.7 µM	2.23	Bromodomain protein regulating differentiation. Nuclear, structurally tractable, and analogous to known PROTAC targets like BRD4.	LogP compatible with permeability; hydrophobic pocket favors linker attachment.
	(**81**)	IC_50_ = 8.4 µM *K*_d_ = 4.0 µM	3.27		
Cruzain	(**91**)	IC_50_ = 0.081 nM	5.04	Major protease involved in immune evasion and differentiation. Immature form is cytosolic. Resistance reported; ligands available.	Excellent potency, but high LogP may limit solubility and selectivity. Needs careful linker and E3 ligase optimization.
	(**95**)	IC_50_ = 0.01 µM	5.19		

Abbreviations: BRD4—bromodomain-containing protein 4; Hsp90—heat shock protein 90; TcAkt—*Trypanosoma cruzi* protein kinase B (Akt homolog); TcBDF3—*Trypanosoma cruzi* bromodomain factor 3; TcCYP51—*Trypanosoma cruzi* sterol 14α-demethylase; TcTopoIIα—*Trypanosoma cruzi* DNA topoisomerase II alpha; TcTR—*Trypanosoma cruzi* trypanothione reductase; UPS—ubiquitin–proteasome system; PROTAC—PROteolysis TArgeting Chimera; LogP—octanol–water partition coefficient; IC_50_—half-maximal inhibitory concentration; *K*_i_—inhibition constant; Kd—dissociation constant; Cys477—cysteine at position 477 (targeted covalently in TcTopoIIα).

### 6.2. Comparative Exploration of the Ubiquitination Machinery in T. cruzi and Humans

The UPS plays a central role in protein homeostasis in eukaryotes and is highly conserved between *T. cruzi* and humans. Both organisms share essential components, including ubiquitin, the E1, E2, and E3 enzymes, deubiquitinases (DUBs), and the 26S proteasome. However, the *T. cruzi* UPS exhibits unique features that render it an attractive therapeutic target, given its involvement in critical parasitic processes such as differentiation between trypomastigote and amastigote forms, cell cycle regulation, and oxidative stress response [234,235]. Compared to the human UPS, *T. cruzi* has a reduced system, with approximately 269 identified proteins versus over 1200 in mammals, indicating lower functional redundancy and increasing the feasibility of selective therapeutic targeting [46].

One of the key structural differences lies in the composition of *T. cruzi* E3 ligases, which exhibit significant variations compared to their human counterparts. The parasite’s E3 ligases can be classified into five major categories, as summarized in the table below (Table 15) [46].

A particularly noteworthy ligase is the small PROTAC-influenced RING-type (SPRING) E3 ligase exclusive to *T. cruzi*, which is secreted into the host and functions in the nucleus of human cells, modulating the immune response. This unique interaction suggests that the UPS in *T. cruzi* regulates intrinsic parasitic processes while also influencing the host’s intracellular environment, thereby enhancing infection [236]. Moreover, studies indicate that certain E3 ligases are differentially expressed throughout the parasite’s life cycle, suggesting distinct roles in cell proliferation and differentiation [201].

UPS involvement in cell cycle regulation is another fundamental aspect of parasite survival. Several ubiquitinated proteins are directly linked to G1/S phase progression and mitosis, making the inhibition of specific E3 complexes a potential strategy for halting parasite replication [237]. UPS-mediated oxidative stress response regulation plays a critical role in *T. cruzi* survival within host cells, allowing for adaptation to the hostile intracellular microenvironment [238].

Building on this functional relevance, the unique features of the parasite UPS have raised interest in identifying parasite-specific E3 ligases that could serve as entry points for TPD strategies. In particular, examining whether any *T. cruzi* orthologs correspond to human E3 ligases commonly used in PROTAC design—such as cereblon (CRBN)—is essential for assessing the feasibility of this approach [229].

Such structural divergence is particularly important when considering thalidomide-based PROTACs, which are widely used to recruit CRBN in mammalian systems [239,240]; yet, they may prove unsuitable in trypanosomatids. In *T. cruzi*, no experimental evidence currently supports the ability of CRBN-like to bind thalidomide, and key structural differences suggest that canonical CRL4^CRBN^ complex formation may not occur. Indeed, recent structural models suggest that the thalidomide-binding pocket in TcCRBN-like is likely collapsed or non-functional, reducing the potential for ligand engagement. These limitations underscore the need to identify alternative ligands and adaptor interfaces compatible with parasite-specific E3 ligases [237].

From a therapeutic perspective, an alternative strategy to developing TrypPROTACs would be to exploit the human ubiquitin–proteasome system (UPS) by employing well-characterized human E3 ligases such as CRBN, Von Hippel–Lindau (VHL), mouse double minute 2 homolog (MDM2), or inhibitor of apoptosis proteins (IAPs) [201]. This approach, while less specific to *T. cruzi*, bypasses the uncertainties related to parasite E3 ligases—such as the questionable suitability of CRBN-like—and could target essential proteins involved in both parasite viability and host cell cycle regulation. However, a major challenge would be achieving selectivity, as a PROTAC designed to harness the human UPS might induce off-target effects in host cells [241].

In this context, targeting proteins shared between *T. cruzi* and the host could be a viable strategy. Proteins involved in proteasome regulation, oxidative stress metabolism—such as nuclear factor erythroid 2–related factor 2 (NRF2) and Kelch-like ECH-associated protein 1 (Keap1)—or cell cycle pathways could be leveraged to inhibit parasite proliferation indirectly [242,243]. Additionally, human UPS-targeting PROTACs could be refined for selective delivery using specialized delivery systems such as functionalized nanoparticles, ensuring preferential accumulation in infected cells [244].

Alternatively, the SCF complex (Skp1–Cullin1–F-box), one of the most versatile E3 ligases, has been shown to be functional in *T. brucei*. In a study by Ishii and Akiyoshi (2022), the deGradFP system successfully degraded both nuclear (kinetochore kinesin 3, KKT3) and cytoplasmic (secretory protein 31, SEC31) proteins using an F-box-based approach, suggesting that endogenous Skp1–Cullin1–F-box (SCF) ligases are conserved and active in trypanosomatids [245]. This proof-of-concept strengthens the rationale for investigating F-box ligases in *T. cruzi* as feasible targets for TrypPROTAC strategies. Moreover, Danazumi et al. (2023) identified at least seven distinct CRL ligases in *T. cruzi*, including a TcCRBN-like member, although none have been biochemically validated [201]. These findings reinforce the need for functional studies to confirm the activity and ligandability of parasite E3 ligases.

While TrypPROTACs represent a more specific and rational approach for Chagas disease treatment, PROTACs based on the human UPS offer an interesting alternative, particularly if designed to target metabolic pathways that impair parasite viability without inducing systemic toxicity. Nonetheless, these approaches must contend with the limited structural and functional knowledge of parasite E3 ligases and the uncertain suitability of canonical ligands such as thalidomide-like derivatives for these systems [201]. Insights from the current literature highlight that, although the machinery for TPD exists in trypanosomatids, its therapeutic exploitation is still at an exploratory stage, requiring a more thorough understanding of E3 ligase diversity, structure, and function in these organisms [46,229,235]. The exploration of combined approaches may provide new perspectives for the development of innovative therapies against *T. cruzi*, positioning this field at the forefront of medicinal chemistry and TPD research.

### 6.3. Rational Linker Design in PROTACs

Linkers in PROTACs play a central role in modulating pharmacokinetic and pharmacodynamic properties, influencing ternary complex formation, target selectivity, cell permeability, and metabolic stability. Their structural and physicochemical characteristics must be carefully optimized to ensure efficient protein degradation while maintaining favorable drug-like properties [246,247]. This aspect becomes even more critical in *T. cruzi*, where membrane composition and intracellular compartmentalization impose additional barriers to effective drug delivery.

As molecular connectors between the POI ligand and the E3 ligase recruiter, linkers must provide the appropriate spatial arrangement for productive ternary complex formation. Flexible linkers, such as alkyl chains or PEG-based moieties, offer conformational adaptability, which can facilitate target engagement. However, excessive flexibility may lead to entropic penalties, reduced selectivity, and an increase in molecular weight and topological polar surface area (TPSA), negatively impacting permeability [246,248]. In contrast, rigid linkers, including heterocyclic and cyclic scaffolds such as triazoles, piperazines, or fused bicyclic systems, impose conformational constraints that can enhance target specificity, improve metabolic stability, and reduce PSA, thereby favoring passive membrane diffusion [249,250].

Permeability remains one of the major challenges in PROTAC development, particularly for intracellular targets. Linkers capable of inducing intramolecular folding, forming hydrogen bonds, or engaging in π-π stacking can minimize solvent-exposed polarity, enhancing cell penetration [246,248,251]. Structural modifications such as amide-to-ester substitutions or fluorine incorporation can further optimize physicochemical properties without significantly compromising solubility [251]. In the case of *T. cruzi*, the parasite’s ergosterol-rich membrane demands a precise balance between hydrophilic and hydrophobic properties. While increased lipophilicity may facilitate membrane diffusion, excessive hydrophobicity can lead to aggregation, reduced solubility, and off-target effects [252].

A rational approach to PROTAC linker design should integrate computational and experimental strategies, including molecular dynamics simulations, permeability modeling, and SAR studies [249,253]. By prioritizing linkers that reduce PSA, optimize intramolecular interactions, and strike a balance between flexibility and rigidity, it is possible to develop PROTACs with enhanced bioavailability and therapeutic potential against *T. cruzi* and other kinetoplastid parasites (Table 16).

### 6.4. Experimental Validation of TrypPROTACs

**Figure 6:**
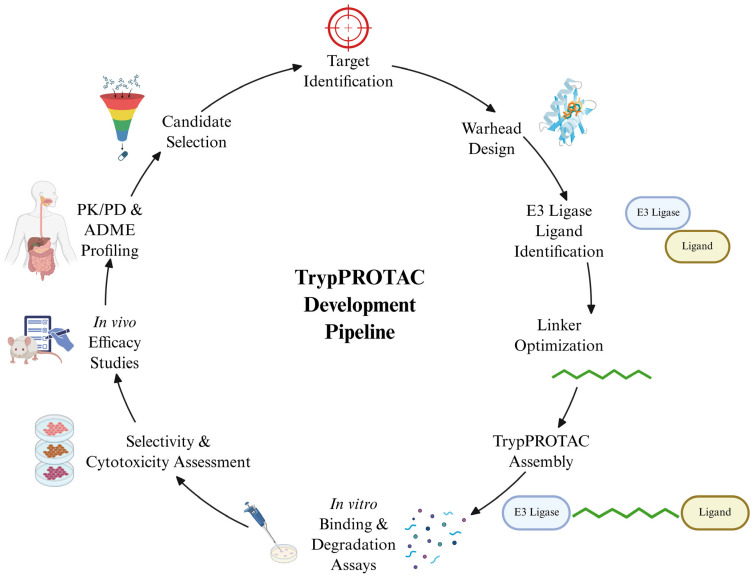
Sequential pipeline for the rational development of TrypPROTACs targeting *T. cruzi* proteins. Created in BioRender. Dos santos, J. (2025) https://biorender.com/r22f256, accessed on 18 May 2025.

The experimental validation of TrypPROTACs involves a coherent sequence of methodologies aimed at confirming target engagement, proteasome-dependent degradation, and antiparasitic efficacy in *T. cruzi*-infected mammalian cells (Figure 6). A critical initial parameter is the intracellular free fraction of the compound, which is typically determined by equilibrium dialysis followed by quantification via liquid chromatography–tandem mass spectrometry (LC–MS/MS). This analysis provides essential information on the bioavailable portion of the molecule within the cellular environment, which is a particularly relevant metric given the physicochemical properties of PROTACs, which often exhibit high molecular weight and polarity, thereby limiting passive permeability [254].

Target engagement is commonly assessed through the Cellular Thermal Shift Assay (CETSA), which detects ligand-induced stabilization of the POI across a thermal gradient [255]. Alternatively, bioluminescence resonance energy transfer-based assays (NanoBRET) permit real-time, live-cell monitoring of target binding by exploiting energy transfer between nanoluciferase (NanoLuc)-tagged proteins and fluorescent tracers, offering high temporal resolution and sensitivity [254,256]. These methodologies are essential to confirm target engagement and binding specificity in live-cell systems.

The formation of a productive ternary complex, essential for successful ubiquitin transfer, is typically monitored using bioluminescent complementation-based assays such as Lumit Immunoassays, which employ NanoBiT (nanoluciferase binary technology) to generate luminescent signals upon ternary complex formation. These platforms are compatible with titration series, competition binding formats, and high-throughput screening campaigns [254]. This strategy enables the quantitative evaluation of cooperativity and the identification of the hook effect.

The subsequent confirmation of target degradation is generally performed by Western blotting, quantitative mass spectrometry, or HiBiT-based assays (high-affinity peptide tags for luminescence detection), depending on the available cellular systems. The inclusion of proteasome inhibitors such as MG132 (Carbobenzoxy-Leu-Leu-leucinal) is essential to confirm that degradation is mediated by the UPS [255,257,258]. Additionally, TUBE-based assays (tandem ubiquitin-binding entities) provide a sensitive and specific method for detecting the polyubiquitination of endogenous proteins, further validating PROTAC-induced degradation pathways [258].

Antiparasitic efficacy is determined in *T. cruzi*-infected host cells by quantifying intracellular parasite load using quantitative polymerase chain reaction (qPCR) targeting kinetoplast or satellite DNA, enzymatic assays with *lacZ*-expressing parasite strains, or fluorescence-based imaging in reporter-expressing lines. These assays support the determination of EC_50_ values under physiologically relevant conditions [256,257,259]. Cytotoxicity in uninfected host cells, including Vero, L6, or C2C12 lines, is evaluated via established viability assays such as MTT (3-(4,5-Dimethylthiazol-2-yl)-2,5-diphenyltetrazolium bromide assay), Alamar Blue, or CellTiter-Glo (luminescent cell viability assay based on ATP quantification). The SI, calculated as the CC_50_/EC_50_ ratio, provides a quantitative measure of the therapeutic window and is essential for compound prioritization.

Collectively, this rigorous experimental cascade constitutes a mechanistically informed and translationally relevant framework for the comprehensive validation of TrypPROTACs. By integrating target engagement, ternary complex formation, proteasome dependency, and phenotypic response, the approach facilitates rational candidate progression toward preclinical evaluation [257,259,260].

## 7. Future Perspectives and Conclusions

The application of TPD via PROTACs to *T. cruzi* represents a paradigm shift in antiparasitic drug discovery. Unlike conventional inhibitors that transiently block enzymatic function, TrypPROTACs are designed to catalytically and selectively eliminate essential parasite proteins, potentially resulting in sustained pharmacodynamic effects and reduced dosing frequency. By hijacking the UPS, this approach enables the degradation of previously “undruggable” targets and offers a promising route to overcome classical resistance mechanisms.

However, the development of TrypPROTACs remains at an early stage and is challenged by both biological and chemical limitations. Chief among these is the limited understanding of the parasite’s ubiquitination machinery, particularly its E3 ligase repertoire. Although genomic annotations suggest the presence of CRL-type ligases and putative orthologs of DDB1, CUL4, and Rbx1 (RING-box protein), their structural and functional properties remain largely uncharacterized, hindering the rational design of parasite-specific E3 ligase binders. This knowledge gap compromises the formation of stable ternary complexes, a critical prerequisite for efficient target degradation.

In addition, the design of effective linkers and warheads must overcome several pharmacokinetic and pharmacodynamic constraints. PROTACs typically exhibit high molecular weights, poor aqueous solubility, and limited membrane permeability, all of which may impair intracellular accumulation, particularly within the cytoplasmic and nuclear compartments where key *T. cruzi* targets such as bromodomain-containing proteins (e.g., TcBDF3), cruzain, and topoisomerases reside. Recent advances in linker chemistry, including the use of rigidified spacers, modulation of lipophilicity, and polar surface area minimization, have shown promise in mitigating these issues.

Importantly, representative ligands for validated *T. cruzi* targets—including bromodomain inhibitors, cruzain binders, and topoisomerase ligands—have already been identified, providing a strong foundation for the rational construction of TrypPROTACs. The development of parasite-specific degraders may also offer enhanced selectivity over host proteins, thereby reducing the likelihood of adverse effects. This consideration is particularly relevant in the context of Chagas disease, where current therapies suffer from poor tolerability and high toxicity.

Beyond classical PROTACs, alternative degradation strategies may offer valuable avenues for therapeutic innovation. Approaches such as molecular glues, LYTACs for extracellular protein degradation, and homo-PROTACs that dimerize parasite proteins could be adapted to *T. cruzi*, especially in scenarios where traditional E3 ligase recruitment proves unfeasible or intracellular delivery is compromised.

Despite encouraging progress, experimental validation remains a critical bottleneck. The adaptation of degradation-specific assays—such as CETSA, NanoBRET, and global proteomics—to the biological context of *T. cruzi* will be essential to accelerate discovery and confirm target engagement. Ultimately, the successful development of TrypPROTACs will depend on the integration of medicinal chemistry, parasitology, chemical biology, and system-level validation strategies. If these challenges can be addressed, then TrypPROTACs may usher in a new generation of antiparasitic agents characterized by improved selectivity, durability, and therapeutic relevance.

## Figures and Tables

**Table 1:** Selected inhibitors targeting the ergosterol and isoprenoid biosynthesis pathways in *T. cruzi*.

Compound	Structure	Affinity Data	LogP	Reference
**1**	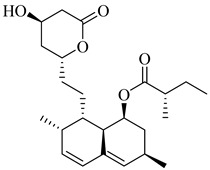	IC_50_ = 0.3 μM (*T. cruzi* epimastigote form)	4.1955	[76]
**2**	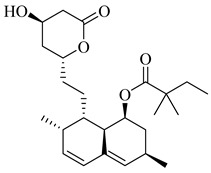	IC_50_ = 0.4 μM (*T. cruzi* epimastigote form)	4.5856	[76]
**3**	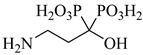	*K*_i_ = 2.02 µM IC_50_ = 1.08 µM (TcFPPS)	−1.6633	[70]
**4**	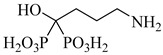	*K*_i_ = 1.04 µM IC_50_ = 0.77 µM (TcFPPS)	−1.2732	[70]
**5**	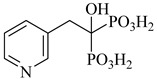	*K*_i_ = 0.032µM IC_50_ = 0.037 µM (TcFPPS)	−0.3744	[70]
**6**	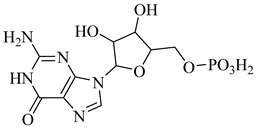	DS = −176.92 kcal/mol (TcFPPS)	−2.5697	[77]
**7** (*R*) **8** (*S*)	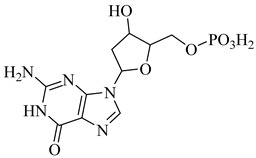	DS = −172.72 kcal/mol (TcFPPS) DS = −172.91 kcal/mol (TcFPPS)	−1.5405	[77]
**9**	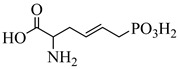	DS = −126.34 kcal/mol (TcFPPS)	−0.4777	[77]
**10**	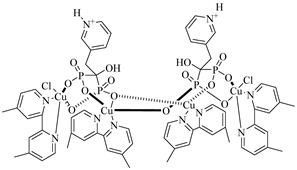	DS = −8.3 kcal/mol (TcFPPS)	—	[78]
**11**	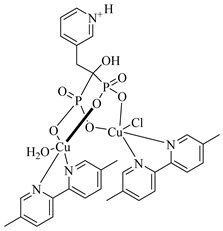	DS = −8.5 kcal/mol (TcFPPS)	—	[78]

Abbreviations: DS = docking score; TcFPPS = *Trypanosoma cruzi* farnesyl pyrophosphate synthase; IC_50_ = half-maximal inhibitory concentration; *K_i_* = inhibition constant; LogP = octanol–water partition coefficient.

**Table 2:** Potent inhibitors of TcSQS: enzymatic and in vitro/in vivo efficacy data.

Compound	Structure	Affinity Data	LogP	Reference
**12**	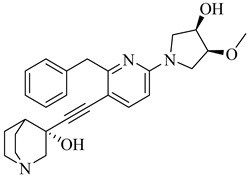	IC_50_ = 6.9 nM (TcSQS)	0.8518	[80]
**13**	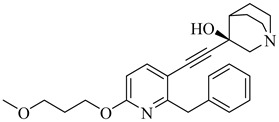	IC_50_ = 6.4 nM (TcSQS)	2.8959	[80]
**14**	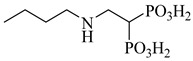	IC_50_ = 39 nM (TcSQS)	0.0576	[81]
**15**	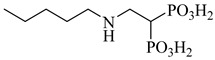	IC_50_ = 5 nM (TcSQS)	0.4477	[81]
**16**	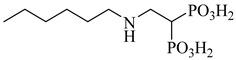	IC_50_ = 21.4 nM (TcSQS)	0.8378	[81]
**17**	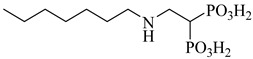	IC_50_ = 11.9 nM (TcSQS)	1.2279	[81]
**18**	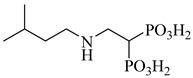	IC_50_ = 22 nM (TcSQS)	0.3036	[81]
**19**	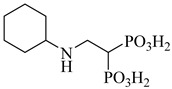	IC_50_ = 30 nM (TcSQS)	0.5902	[81]

Abbreviations: TcSQS = *T. cruzi* squalene synthase; IC_50_ = half-maximal inhibitory concentration; LogP = octanol–water partition coefficient.

**Table 5:** Functional and pharmacological characterization of TcGAPDH in *T. cruzi*.

Compound	Structure	Affinity Data	LogP	Reference
**38**	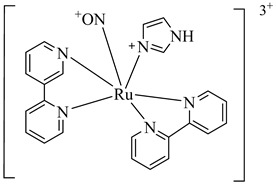	IC_50_ = 89 µM (TcGAPDH)	—	[105]
**39**	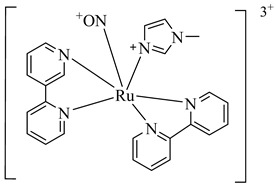	IC_50_ = 97 µM (TcGAPDH)	—	[105]
**40**	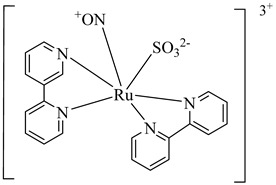	IC_50_ = 153 µM (TcGAPDH)	—	[105]
**41**	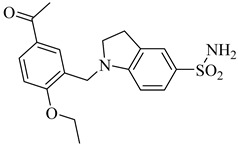	*K*_m_ = 8.24 µM (TcGAPDH)	2.498	[106]
**42**	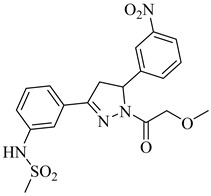	*K*_m_ = 11.60 µM (TcGAPDH)	2.2904	[106]
**43**	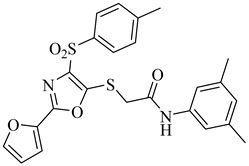	*K*_m_ = 11.61 µM (TcGAPDH)	5.42346	[106]

Abbreviations: TcGAPDH = *T. cruzi* glyceraldehyde-3-phosphate dehydrogenase; IC_50_ = half-maximal inhibitory concentration; *K_m_* = Michaelis constant; LogP = octanol–water partition coefficient.

**Table 11:** Recent advances in the development of inhibitors targeting TcTS to disrupt host–parasite interactions and immune evasion mechanisms in *T. cruzi*.

Compound	Structure	Affinity Data	LogP	Reference
**83**	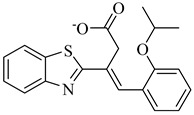	92% inhibition (at 1 mM) (TcTS) IC_50_ = 0.29 mM (TcTS)	3.7641	[168]
**84**	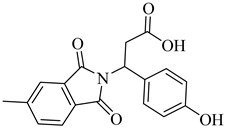	82.5% inhibition (at 1 mM) (TcTS)	2.51262	[169]
**85**	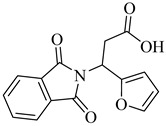	76.5% inhibition (at 1 mM) (TcTS)	2.0916	[169]
**86**	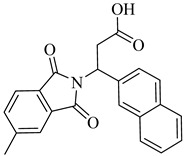	86.9% inhibition (at 1 mM) (TcTS) DS = −11.1 kcal/mol (TcTS)	3.96022	[169]
**87**	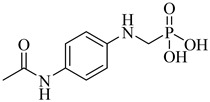	IC_50_ = 0.23 mM (at 1 mM) (TcTS) *K*_i_ = 190 µM (TcTS)	1.1921	[169]
**88**	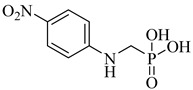	IC_50_ = 0.21 Mm (at 1 mM) (TcTS) *K*_i_ = 140 µM (TcTS)	1.1419	[169]
**89**	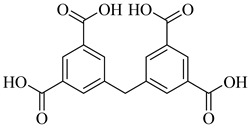	87.6% inhibition (at 1 mM) (TcTS) DS = −9.6 kcal/mol (TcTS)	2.0702	[170]

Abbreviations: DS = docking score; TcTS = *T. cruzi* trans-sialidase; IC_50_ = half-maximal inhibitory concentration; *K_i_* = inhibition constant; LogP = octanol–water partition coefficient.

**Table 12:** Discovery and optimization of cruzain inhibitors targeting cysteine protease activity in *T. cruzi* for Chagas disease therapy.

Compound	Structure	Affinity Data	LogP	Reference
**90**	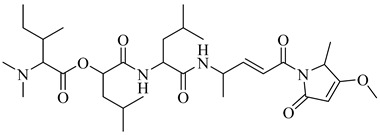	IC_50_ = 1.3 nM (cruzain)	2.8	[180]
**91**	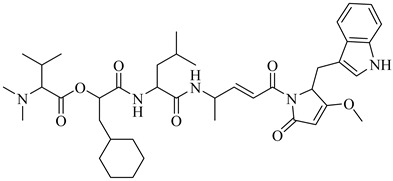	IC_50_ = 0.081 nM (cruzain)	5.0383	[180]
**92**	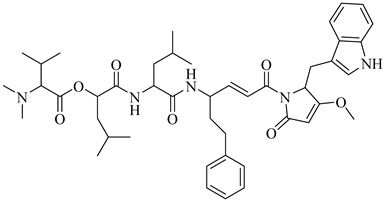	IC_50_ = 0.09 nM (cruzain)	5.7269	[180]
**93**	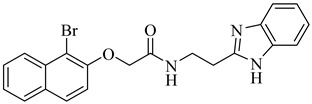	IC_50_ = 0.2 µM (cruzain)	4.2163	[181]
**94**	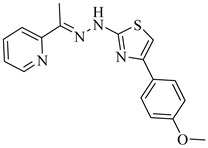	IC_50_ = 0.04 µM (cruzain)	4.0498	[182]
**95**	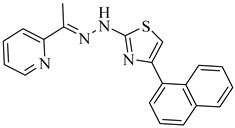	IC_50_ = 0.01 µM (cruzain)	5.1944	[182]

Abbreviations: IC_50_ = half-maximal inhibitory concentration; LogP = octanol–water partition coefficient.

**Table 13:** Novel nitroaromatic scaffolds bioactivated by TcNTR in *T. cruzi* with strategies focused on enhancing selectivity and overcoming drug resistance in Chagas disease.

Compound	Structure	Affinity Data	LogP	Reference
**96**	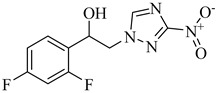	IC_50_ = 0.39 µM (*T. cruzi)*	1.1981	[187]
**97**	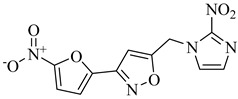	*K*_obs_ = 1.27 s^−1^ (at 25 µM) (TcNTR)	1.9958	[188]
**98**	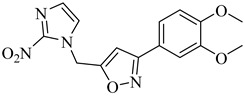	*K*_obs_ = 0.82 s^−1^ (at 25 µM) (TcNTR)	2.5118	[188]
**99**	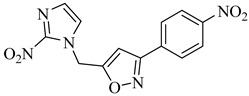	*K*_obs_ = 0.68 s^−1^ (at 25 µM) (TcNTR)	2.4028	[188]
**100**	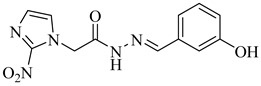	*K*_obs_ = 0.32 (at 25 µM) *K*_obs_ = 0.47 (at 50 µM) (TcNTR)	0.6472	[189]

Abbreviations: IC_50_ = half-maximal inhibitory concentration; TcNTR = *T. cruzi* nitroreductase type I; *K_obs_* = observed rate constant; LogP = octanol–water partition coefficient.

**Table 15:** Classification and structural characteristics of predicted E3 ubiquitin ligases in *T. cruzi*.

E3 Ligase Class	Characteristics
RING-domain E3	Largest class in *T. cruzi* (67 predicted proteins), mediates direct ubiquitin transfer without a thioester intermediate.
Cullin-RING Ligases (CRLs)	A subset of RING ligases, forming multiprotein complexes essential for regulated protein degradation. *T. cruzi* possesses 13 CRLs.
F-Box E3 Ligases	Associated with the SCF complex (Skp1-Cullin1-F-box), involved in substrate selection for degradation, with eight identified proteins.
U-Box E3 Ligases	Structurally similar to RING ligases yet lacking metal coordination, these proteins are involved in protein quality control, with five members identified to date.
HECT-domain E3	Transfers ubiquitin via a thioester intermediate. Sixteen proteins have been identified, with additional domains such as SPRY and ZnF RBZ.

Abbreviations: CRLs—Cullin-RING ligases; E3—E3 ubiquitin ligase; F-box—F-box domain-containing protein; HECT—homologous to E6AP C-Terminus; RING—really interesting new gene; SCF—Skp1–Cullin1–F-box protein complex; Skp1—S-phase kinase-associated protein 1; SPRY—SPla and the RYanodine receptor domain; U-box—ubiquitin ligase box; ZnF RBZ—zinc finger ranBP2-type domain.

**Table 16:** Linker types used in PROTAC design: structural features, permeability impact, and considerations for *T. cruzi*.

Linker Type	Examples	Structural Features	Impact on Permeability	Relevance for *T. cruzi*
Flexible	Alkyl chains (-CH_2_-)	High conformational freedom, adaptable.	May increase entropic cost and reduce selectivity.	Facilitates complex formation, although it requires optimization to prevent structural collapse.
Hydrophilic	PEG (-OCH_2_CH_2_-)	Enhances aqueous solubility, increases TPSA.	Often reduces passive diffusion.	Useful for solubility tuning; however, excessive polarity should be avoided.
Rigid	Triazoles, aromatic linkers	Conformationally constrained, stabilizes ternary complex.	Enhances selectivity; however, it may restrict flexibility.	Ideal for improving protein–protein interactions and metabolic stability.
Semi-Rigid	Piperazine, piperidine	Balances flexibility and rigidity, reduces PSA.	Promotes intramolecular folding, enhances passive diffusion.	Strong candidate for modulating permeability and metabolic stability.
Lipophilic	Fused rings, spirocycles	Reduces PSA, increases membrane permeability.	Enhances passive transport; however, it may lead to aggregation.	Can optimize intracellular access if carefully tuned.

Abbreviations: PEG—polyethylene glycol; TPSA—topological polar surface area; PSA—polar surface area.

## Data Availability

No new data were created or analyzed in this study. Data sharing is not applicable.

## Abbreviations

The following abbreviations are used in this manuscript:

6PGDH	6-Phosphogluconate dehydrogenase
24-SMT	Δ24(25)-sterol methyltransferase
AEK1	AGC essential kinase 1
Akt	akt-like kinase
BDF3	bromodomain Factor 3
BRD4	bromodomain-containing protein 4
BZN	benznidazole
CC_50_	half-maximal cytotoxic concentration
CDK4/6	cyclin-dependent kinases 4 and 6
CETSA	Cellular Thermal Shift Assay
ClpC-ClpP complex	caseinolytic protease system
CoMFA	Comparative Molecular Field Analysis
CPx	cytosolic tryparedoxin peroxidase
CRBN	Cereblon
CRL	Cullin-RING Ligase
CYP51	sterol 14α-demethylase
DHFR	dihydrofolate reductase
DHFR–TS	dihydrofolate reductase–thymidylate synthase (bifunctional)
DHODH	dihydroorotate dehydrogenase
DS	docking score
DUB	deubiquitinase
EC_50_	half-maximal effective concentration
EGFR	epidermal growth factor receptor
FMN	flavin mononucleotide
FPP	farnesyl diphosphate synthase
FPPS	farnesylpyrophosphate synthase
FTase	farnesyltransferase
G3P	glyceraldehyde-3-phosphate
G6P	glucose-6-phosphate
GAPDH	glyceraldehyde-3-phosphate dehydrogenase
GI_50_	half-maximal growth inhibitory concentration
GlcK	glucokinase
GR	glutathione reductase
gRNAs	guide RNAs
HER2	human epidermal growth factor receptor 2
HGPRT	hypoxanthine-guanine phosphoribosyltransferase
HGXPRT	hypoxanthine-guanine-xanthine phosphoribosyltransferase
HiBiT	High-affinity Bioluminescent Tag
HIV-1	Human Immunodeficiency Virus type 1
HMG-CoA	3-hydroxy-3-methylglutaryl coenzyme A
HPV	Human Papillomavirus
HQSAR	hologram quantitative structure–activity relationship
Hsps	heat shock proteins
HxK	hexokinase
IAP	inhibitor of apoptosis protein
IC_50_	half-maximal inhibitory concentration
IPC	inositolphosphorylceramide
kDNA	kinetoplast DNA
*K*_d_	dissociation constant
*Ki*	inhibition constant
*Ki_app_*	apparent inhibition constant
*K_m_*	Michaelis–Menten constant
Keap1	Kelch-like ECH-associated protein 1
Kobs	observed rate constant
KKT3	kinetochore kinesin 3
*L. mexicana*	*Leishmania mexicana*
LC_50_	half-maximal lethal concentration
LC–MS/MS	liquid chromatography–tandem mass spectrometry
LogP	logarithm of the partition coefficient (octanol/water)
LS	lanosterol synthase
MDM2	mouse double minute 2 homolog
MPx	mitochondrial tryparedoxin peroxidase
MuNANA	4-methylumbelliferyl-*N*-acetylneuraminic acid assay
MYC	myelocytomatosis oncogene
NADPH	nicotinamide adenine dinucleotide phosphate (reduced form)
NanoBiT	nanoluciferase binary technology
NanoBRET	bioluminescence resonance energy transfer–based assays
NanoLuc	nanoluciferase
NFX	nifurtimox
NMR	Nuclear Magnetic Resonance
NRF2	nuclear factor erythroid 2–related factor 2
NTR	nitroreductase
OSC	oxidosqualene cyclase
PFK	phosphofructokinase
PH	pleckstrin homology
POI	protein of interest
PROTAC	proteolysis-targeting chimeras
PRPP	5-phospho-α-D-ribose 1-pyrophosphate
PRT	purine phosphoribosyltransferase
PTR	pteridine reductase
QFIT	quantitative pharmacophore fit value
qPCR	quantitative polymerase chain reaction
QSAR	quantitative structure–activity relationship
Rbx1	RING-box protein
ROS	reactive oxygen species
RTK	receptor tyrosine kinase
SAPA	shed acute-phase antigen
SAR	structure–activity relationship
SCF complex	Skp1–Cullin1–F-box
SE	squalene epoxidase
SEC31	secretory protein 31
SI	selectivity index
SOD	superoxide dismutase
SQS	squalene synthase
SPRING	small PROTAC-influenced RING-type
*T. brucei*	*Trypanosoma brucei*
*T. cruzi*	*Trypanosoma cruzi*
TMQ	trimetrexate
Topo3α	topoisomerase 3α
TPD	targeted protein degradation
TPSA	topological polar surface area
TR	trypanothione reductase
TS	trans-sialidase
TryS	trypanothione synthetase
TXNI	tryparedoxin I
TXNPx	tryparedoxin peroxidase
UPS	ubiquitin-proteasome system
VHL	Von Hippel-Lindau
